# Replicative DNA Polymerase δ but Not ε Proofreads Errors in *Cis* and in *Trans*

**DOI:** 10.1371/journal.pgen.1005049

**Published:** 2015-03-05

**Authors:** Carrie L. Flood, Gina P. Rodriguez, Gaobin Bao, Arthur H. Shockley, Yoke Wah Kow, Gray F. Crouse

**Affiliations:** 1 Department of Biology, Emory University, Atlanta, Georgia, United States of America; 2 Department of Radiation Oncology, Emory University, Atlanta, Georgia, United States of America; 3 Winship Cancer Institute, Emory University, Atlanta, Georgia, United States of America; University of Iowa Carver College of Medicine, UNITED STATES

## Abstract

It is now well established that in yeast, and likely most eukaryotic organisms, initial DNA replication of the leading strand is by DNA polymerase ε and of the lagging strand by DNA polymerase δ. However, the role of Pol δ in replication of the leading strand is uncertain. In this work, we use a reporter system in *Saccharomyces cerevisiae* to measure mutation rates at specific base pairs in order to determine the effect of heterozygous or homozygous proofreading-defective mutants of either Pol ε or Pol δ in diploid strains. We find that wild-type Pol ε molecules cannot proofread errors created by proofreading-defective Pol ε molecules, whereas Pol δ can not only proofread errors created by proofreading-defective Pol δ molecules, but can also proofread errors created by Pol ε-defective molecules. These results suggest that any interruption in DNA synthesis on the leading strand is likely to result in completion by Pol δ and also explain the higher mutation rates observed in Pol δ-proofreading mutants compared to Pol ε-proofreading defective mutants. For strains reverting via AT→GC, TA→GC, CG→AT, and GC→AT mutations, we find in addition a strong effect of gene orientation on mutation rate in proofreading-defective strains and demonstrate that much of this orientation dependence is due to differential efficiencies of mispair elongation. We also find that a 3′-terminal 8 oxoG, unlike a 3′-terminal G, is efficiently extended opposite an A and is not subject to proofreading. Proofreading mutations have been shown to result in tumor formation in both mice and humans; the results presented here can help explain the properties exhibited by those proofreading mutants.

## Author Summary

Many DNA polymerases are able to proofread their errors: after incorporation of a wrong base, the resulting mispair invokes an exonuclease activity of the polymerase that removes the mispaired base and allows replication to continue. Elimination of the proofreading activity thus results in much higher mutation rates. We demonstrate that the two major replicative DNA polymerases in yeast, Pol δ and Pol ε, have different proofreading abilities. In diploid cells, Pol ε is not able to proofread errors created by other Pol ε molecules, whereas Pol δ can proofread not only errors created by other Pol δ molecules but also errors created by Pol ε molecules. We also find that mispaired bases not corrected by proofreading have much different likelihoods of being extended, depending on the particular base-base mismatch. In humans, defects in Pol δ or Pol ε proofreading can lead to cancer, and these results help explain the formation of those tumors and the finding that Pol ε mutants seem to be found as frequently, or more so, in human tumors as Pol δ mutants.

## Introduction

Unlike prokaryotes, eukaryotic cells have multiple DNA polymerases involved in chromosomal replication. It was first demonstrated in *Saccharomyces cerevisiae* [1] and then in human cells [2] that Pol α, Pol δ, and Pol ε were necessary for normal replication. It was subsequently found that two of these polymerases, Pol δ and Pol ε, had 3′ to 5′ exonuclease proofreading activities that could be inactivated to yield proofreading defective enzymes [3–5]. The Pol α-primase complex initiates DNA replication with short RNA primers followed by limited elongation by Pol α; this initiation takes place for each Okazaki fragment and is likely the case for initial initiation of the leading strand as well [6]. Using the two proofreading mutants and analysis of various mutational spectra, it was proposed that leading and lagging strands of replication were each replicated primarily by only one of the two polymerases, Pol δ and Pol ε, [7–9]. At that point, it was not possible to determine which of the polymerases was responsible for each of the replication strands. The use of mutations in each of the DNA polymerases that decrease their fidelity has proven very useful in analyzing their roles in replication. It was suggested that Pol δ, but not Pol ε, could proofread errors created by Pol α [10], supporting a model in which lagging strand synthesis was performed by Pol δ. Mutator alleles of Pol ε were consistent with its role in leading strand synthesis [11], and mutator alleles of Pol δ showed its activity in lagging strand synthesis [12]. A later genome-wide analysis using a Pol δ mutator allele again demonstrated that most Pol δ errors were on the lagging strand [13]. Therefore a current model of replication in yeast is that the lagging strand is replicated by Pol δ and the leading strand by Pol ε. The fact that a similar differentiation is observed in the very distantly related yeast *Schizosaccharomyces pombe* has led to the suggestion that this model is likely true for at least most eukaryotes [14].

One major issue in understanding yeast DNA replication has been the extent to which the leading strand is replicated only by Pol ε. It was found that the catalytic activity of Pol ε is not essential [15], demonstrating that Pol δ is in some cases able to replicate both strands. In addition, it has been consistently found that proofreading defective alleles of Pol ε have a much weaker mutator phenotype than do proofreading defective alleles of Pol δ [6–8,16–18]. Such results have led to proposals that Pol δ could replicate the leading strand under conditions of dysfunction [19] or could be part of an alternative fork formed after stalling on the leading strand [20]. However, the most comprehensive model of Pol δ involvement in leading strand replication was proposed by Pavlov and Shcherbakova based on an extensive survey of the literature and some of their unpublished work [6]. Their model also has initial synthesis of the leading strand by Pol ε and the lagging strand by Pol δ. They envision a variety of different possibilities for an interruption on the leading strand, including incorporation of an incorrect nucleotide that would be difficult to extend, a lesion on the leading strand, collision with RNA polymerase, or spontaneous dissociation of Pol ε. In any of those cases, they propose that reinitiation would be done by Pol δ and not Pol ε [6].

In addition to proofreading, an extremely important system for maintaining fidelity of replication is the mismatch repair system (MMR). The mismatches that result from incorporation of mispaired bases are recognized in eukaryotes by homologues of the bacterial protein MutS, generally MutSα, a heterodimer of Msh2 and Msh6, and MutSβ, a heterodimer of Msh2 and Msh3 [21–23]. Base-base mismatches are recognized almost entirely by MutSα, although there is evidence for recognition of some base-base mispairs by MutSβ [24]. Insertion and deletion mismatches are recognized by both MutSα and MutSβ, with small loops preferentially recognized by MutSα and larger loops preferentially recognized by MutSβ [21–23] with an additional preference of MutSα for repair of insertion loops and repair of deletion loops by MutSβ [25]. Recognition by MutS homologues is followed by interaction with homologues of MutL, usually MutLα in eukaryotes [21–23]. The newly replicated DNA is excised, followed by resynthesis. The method of MMR strand discrimination is still not completely known, but is likely a result of the endonuclease activity of the MutL homologues and interaction of MMR proteins with PCNA [26–28] as well as the incorporation of ribonucleotides into the newly synthesized strand [29,30]. Given the role of proofreading in eliminating mispairs and the role of MMR in repair of mismatches, one might predict that the two systems would function in the same pathway and would exhibit synergistic interactions, and that is what has been observed [3,6,7,17,18]. Although haploid strains of *S. cerevisiae* defective either in MMR or Pol δ proofreading grow well and are viable, the double mutant is not viable, whereas the corresponding double mutant with a Pol ε proofreading defect is viable [3]. This latter result seems to indicate that Pol ε proofreading plays a lesser role in replication than does Pol δ proofreading.

In addition to proofreading and MMR, a major determinant of replication fidelity is the accuracy of the polymerase itself and its ability to extend mispaired nucleotides [31]. DNA polymerases can vary substantially in their fidelity; both Pol δ and Pol ε are relatively accurate even in the absence of proofreading [32,33]. If a mispair is formed and not corrected by proofreading, there is a wide variability in how well various mispairs can be extended—as measured, for example, *in vitro* with *Taq* DNA polymerase or *E*. *coli* Pol I Klenow fragment [34,35]. The difference in extension efficiency can be explained at least in part by the structure of the mispairs [36].

It has proven difficult to study proofreading *in vivo*. Due to the strong synergism with MMR, any measurement of proofreading in the presence of MMR reveals only those mispairs that escaped MMR. However, proofreading defects coupled with an absence of MMR give mutation rates so high that the resulting strains are sick, even as diploids. The situation is even more complex if one is interested in analyzing the role of each replicative polymerase, as the direction of replication of a given assay region is frequently not known, and thus the mispair leading to mutation is indeterminate. In this work, we make use of a collection of *trp5* mutants, each of which can revert to wild type via only one given base pair change [37]. Those *trp5* alleles are placed in a region with a dependable origin of replication so that for each strain it is known which strand is replicated on the leading strand and which on the lagging strand [37]. In order to examine proofreading in the absence of MMR, we use diploid strains that are hemizygous for the *trp5* mutations. Our results are consistent with replication of the lagging strand by Pol δ and initial replication of the leading strand by Pol ε. However, we find that Pol δ can proofread errors on both leading and lagging strands, including errors created by other proofreading-defective Pol δ molecules, whereas a Pol ε molecule is only able to proofread its own errors. It has been difficult to reconcile the much greater mutator phenotype of Pol δ-proofreading deficient strains compared to Pol ε-proofreading deficient strains with a model of replication in which each of the DNA polymerases is primarily responsible for the replication of one strand of DNA. Our results explain this discrepancy by demonstrating Pol δ proofreading of Pol ε errors on the leading strand. Our finding that reversion rates of diploid strains heterozygous for either proofreading deficiency are similar is consistent with reports that Pol ε-proofreading deficient human cells have a disposition toward tumor formation at least as strong as that of Pol δ-proofreading deficient cells. We find large differences in extension efficiencies of various base-base mismatches and additionally find that an 8-oxoG-A mismatch is extended very efficiently and is not subject to proofreading. The demonstration of greatly varying mismatch extension biases *in vivo* can potentially help explain the striking differences in tumor spectra between mammalian cells deficient in Pol δ proofreading compared to Pol ε.

## Results

Various methods have been used to examine the effects of proofreading and to distinguish replication of the leading and lagging strands. We decided to make use of strains containing mutations in an essential codon of the *TRP5* gene because of the mutation specificity and low background of spontaneous reversions [37]. Although this assay permits analysis of base pair mismatches in only one sequence context, it does allow the study of specific mispairs in ways not previously possible. Because we were interested in studying the effect of proofreading on replication, it was necessary to use strains deficient in MMR, as there is a strong synergism between MMR and proofreading [3,6,7]; in addition, MMR shows replication strand bias, further complicating analysis in the presence of MMR [38,39]. For experiments involving defective MMR, in contrast to almost all previous studies of proofreading we used strains deficient in MutSα (*msh6*) since the overall mutational burden is less in such strains compared to a total deficiency in MMR [18] and there is at most only a slight effect of MutSβ on base pair substitution mutagenesis [24]. Our assumption in using strains deficient only in MutSα was that they would be somewhat healthier than those devoid of all MMR, and that assumption has recently been shown to be true for *pol2–4* strains [40].

Because haploid cells deficient in both Pol δ proofreading and MMR are not viable [6], diploid strains were necessary for all of our studies. We used the well-studied *pol2–4* allele to create Pol ε polymerases devoid of proofreading [4], and the *pol3–5DV* allele to disrupt proofreading in Pol δ [10,41–44]. A strain of opposite mating type, deleted for the *TRP5* gene, was crossed with various *trp5* mutant strains, creating hemizygous *trp5* mutants whose reversion could be measured by plating on media lacking tryptophan.

**Fig 1:**
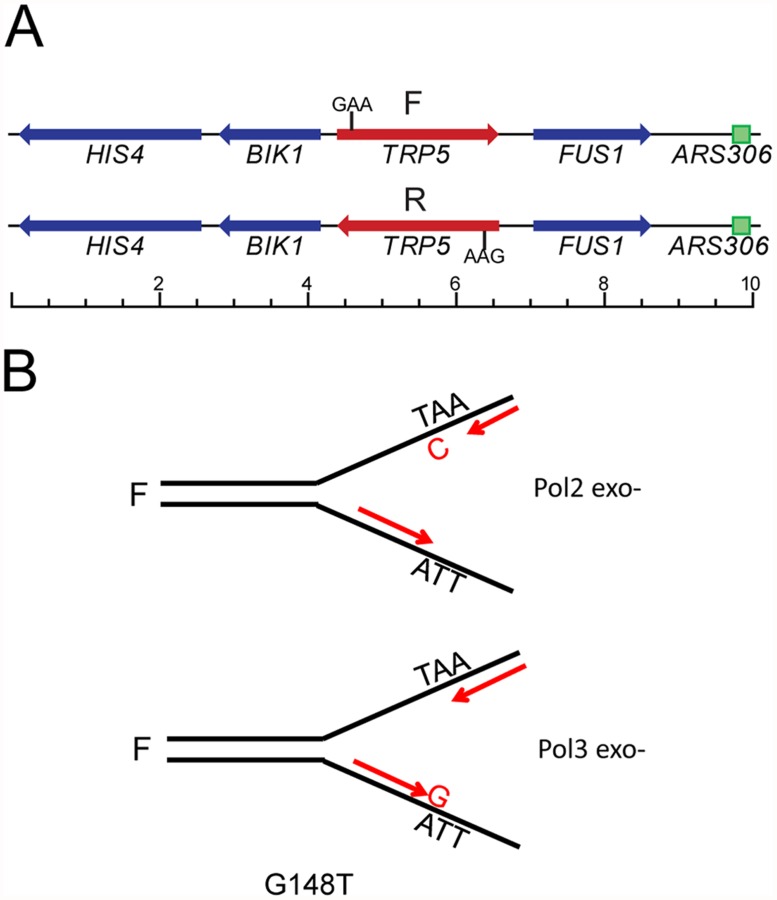
The *trp5* reversion assay in proofreading defective strains. As described previously [37], the *TRP5* gene was deleted from its normal chromosomal location and inserted to replace the *RNQ1* gene on chromosome III in both orientations. (A) The location of the moved *TRP5* gene relative to the *ARS306* origin of replication is shown for both the forward (F) and reverse (R) orientation; scale is marked in kb. The location of the essential GAA codon is also indicated. (B) The *trp5-G148T* allele can only revert via a TA→GC mutation. Assuming that Pol ε replicates the leading strand and Pol δ replicates the lagging strand, the mispair that was responsible for a reversion event can be inferred in any proofreading defective *trp5* mutant. For example, in the F orientation, a proofreading defective Pol ε mutation (Pol2 exo-) will only increase T-C mispairs and not A-G mispairs; similarly a proofreading defective Pol δ mutation (Pol3 exo-) will only increase A-G mispairs and not C-T mispairs.

As shown in Fig. 1A, each of the *trp5* alleles is present in both forward and reverse orientations near a dependable origin of replication (*ARS306*), such that a given DNA sequence will be replicated on the leading strand in one orientation and on the lagging strand in the other orientation [37]. Because of the extremely low reversion rates in wild-type strains, and unlike previously used assays to study proofreading, the observed increase in reversion rates in proofreading-defective strains is due almost entirely to the mispair created on the strand replicated by the proofreading-defective polymerase. A specific example with the *trp5-G148T* allele is illustrated in Fig. 1B. The *trp5-G148T* allele reverts to wild-type via a T-A to G-C mutation which would occur by incorporation of either a T-C or A-G mispair during replication. For most spontaneous revertants, there would be no way to ascertain which mispair had occurred. However, if one assumes a model in which Pol ε, whose catalytic subunit is encoded by the *POL2* gene, replicates the leading strand and Pol δ, whose catalytic subunit is encoded by the *POL3* gene, replicates the lagging strand, the identity of the mispair inducing the reversion can be determined. For strains in the F orientation, reversion in Pol ε-proofreading defective strains would be due to T-C mispairs, while reversion in Pol δ-proofreading defective strains would be due to A-G mispairs. The opposite would be true in strains with the R orientation.

### *trp5-G148T* and *trp5-A149C* alleles display orientation-dependent reversion rates in strains deficient in proofreading and MMR

**Fig 2:**
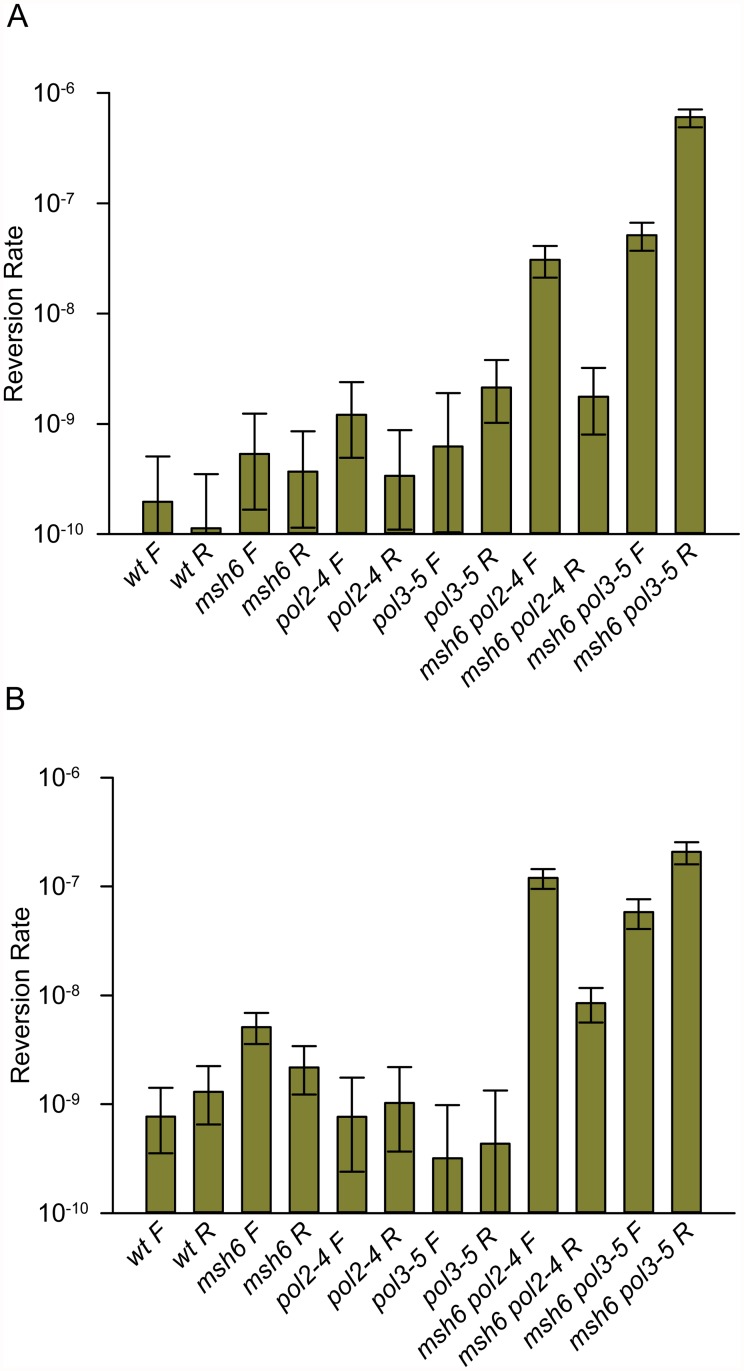
Reversion rates of *trp5-G148T* and *trp5-A149C* diploid strains show major increases only when lacking both MMR and one proofreading activity. Reversion rates of diploid strains with the indicated genotypes were determined as detailed in Materials and Methods. All indicated mutations are homozygous. Error bars represent 95% Confidence Intervals. F and R refer to the orientation of the hemizygous *TRP5* gene relative to the *ARS306* origin of replication. (A) *trp5-G148T* strains; data are given in S1 Table. (B) *trp5-A149C* strains; data are given in S2 Table.

We first measured the rates of spontaneous reversion of the hemizygous *trp5-G148T* allele to the Trp+ phenotype in various genetic backgrounds. In general, the single-mutant reversion rates were not distinguishable from each other or from wild-type, in part due to the very low reversion rates and correspondingly large Confidence Intervals (Fig. 2A and S1 Table). It has been known for many years that defects in proofreading and MMR were synergistic [3,7], and the reversion rates of strains deficient in both MMR and one proofreading activity (Fig. 2A and S1 Table) strongly demonstrate that fact. With one exception (the *trp5-G148T msh6 pol2–4* R strain) the double-mutant reversion rates were one to two orders of magnitude higher than any of the single-mutant reversion rates. Although defects in Pol ε proofreading (*pol2–4*) generally result in a lower mutation rate than defects in Pol δ (*pol3–01* or *pol3–5DV*) [7,43], we observed that in *msh6* strains with the F orientation, the mutation rates were approximately the same (compare *msh6 pol2–4* F with *msh6 pol3–5DV* F in Fig. 2A), whereas they were vastly different in *msh6* strains with the R orientation.

Based on previously reported results, we would have expected that the reversion rates for the *msh6 pol3–5DV* strains to have been much higher than the *msh6 pol2–4* strains in either orientation. Because of the unexpected differences in reversion rates of the double mutants, we repeated the experiments in a second set of diploid strains, containing the hemizygous *trp5-A149C* allele (Fig. 2B and S2 Table). Reversions of the *trp5-A149C* allele occur via the same mismatches as for the above trp5*-G148T* allele, but the bases on the primer and template strands are switched. The pattern of reversion rates in the *trp5-A149C* strains was very similar to that of the *trp5-G148T* strains (compare Fig. 2A to Fig. 2B) with wild-type or single-mutant strains having low and generally similar reversion rates and double-mutant strains having much higher reversion rates. Thus in two independent sets of strains, we observed striking effects of the orientation of the *TRP5* marker gene on reversion rates due to proofreading defects; for *pol2–4 msh6* strains, reversion rates were much higher in the F orientation and for *pol3–5DV msh6* strains reversion rates were much higher in the R orientation.

The effect of *TRP5* orientation on reversion rates of the double mutant strains was both striking and unexpected. These results were uncovered due to the novelty of this assay; previous assays for spontaneous mutations in proofreading mutants have used either forward mutation rates in genes such as *CAN1* or *URA3* that give many different types of mutations [3,7,8,10,16,18,40,41,43,45–50] or reversion assays that usually involve slippage in simple repeats to give frameshifts in the *his7–2* or *hom3–10* alleles or various alleles of *LYS2* [3,7,10,16–18,41,43,45–49,51,52]. Unless the replication direction of a marker gene is known, there is little information to be gained by measuring mutation frequencies of an inverted copy of the gene. With only two exceptions, none of the mutation assays referred to above examined orientation of the marker gene for spontaneous mutation. Those two exceptions were analysis of mutation spectra in a *URA3* gene in both orientations near a defined origin of replication in the chromosome [7] and of the *SUP4-o* gene in both orientations on a yeast plasmid [8]. In both cases, the mutational spectra were different in the two orientations, which was used as an argument that the two polymerases replicated different strands, but there was no analysis of any differences in mutation rates in the two orientations [7,8].

**Fig 3:**
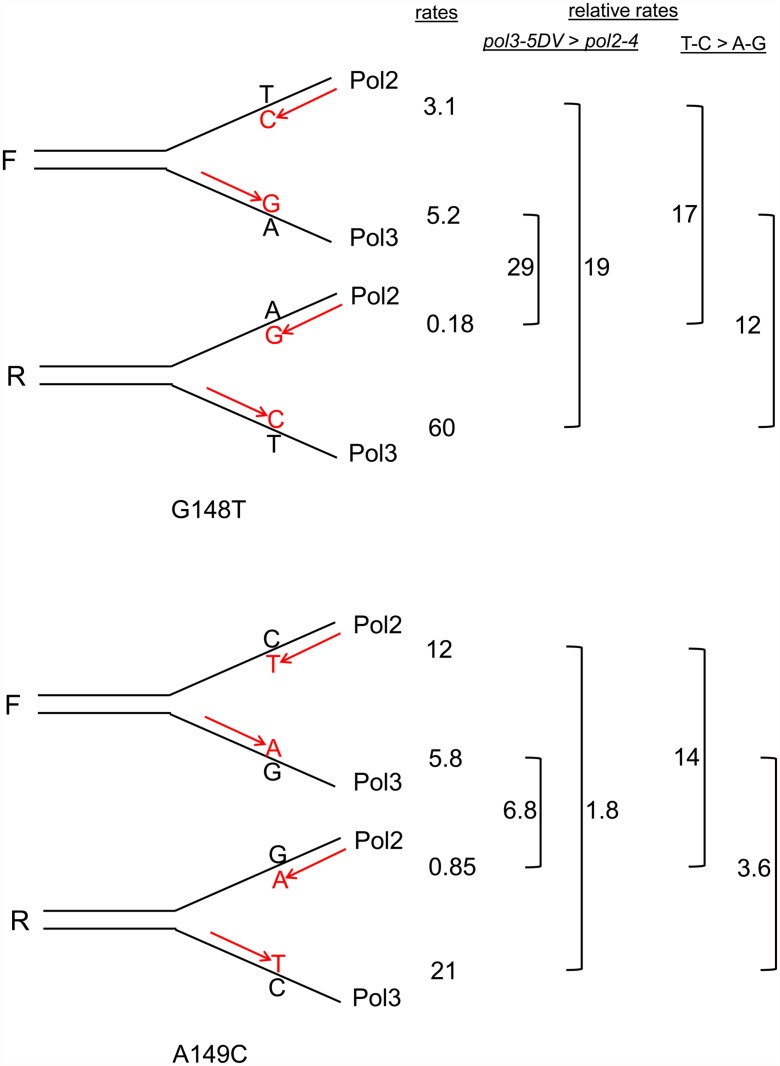
Reversion rates and expected mismatches in proofreading-defective *trp5-G148T* and *trp5-A149C* strains. The mismatches at the replication fork necessary for reversion in the *trp5-G148T* and *trp5-A149C* strains are illustrated in the forward (F) or reverse (R) orientation relative to the *ARS306* origin of replication [37]. New strands are indicated in red, and the inserted base necessary for reversion is also indicated in red. The difference in reversion rates of the proofreading-defective strains is due to increased mispairs on the strand replicated by the appropriate polymerase, the leading strand by Pol ε, whose catalytic subunit is Pol2, and the lagging strand by Pol δ, whose catalytic subunit is Pol3 [12]. To the right of each indicated mispair is the reversion rate of the *msh6* strain (x 10^-8^) that should have increased levels of that mispair (data from Fig. 2 and S1 and S2 Tables). For example, the reversion rate of the *trp5*-*G148T* F *msh6 pol2–4* strain is 3.1. To the right of the reversion rates are comparisons of reversion rates for the same mispair in *msh6 pol3–5DV* versus *msh6 pol2–4* strains and comparisons of reversion rates due to T-C compared to A-G mispairs in either *msh6 pol3–5DV* or *msh6 pol2–4* strains. In every case, the reversion rate for a given mispair is greater in *msh6 pol3–5DV* strains than in *msh6 pol2–4* strains, and the reversion rate due to T-C mispairs is greater than for A-G mispairs.

As discussed above, assuming that the leading strand was replicated by Pol ε and the lagging strand by Pol δ [11,12], it is possible to infer which mispair was increased in each strain. For example, in the *trp5-G148T* strain in the F orientation, a defect in Pol2 proofreading should increase the number of T-C mispairs, but should have no effect on the number of A-G mispairs (see Fig. 1B). For each double mutant strain in Fig. 2, we could associate a reversion rate with the particular mispair that should have led to the reversion event as shown in Fig. 3. We then did two comparisons. For a given mispair, we compared the reversion rate for that mispair in a *pol3–5DV* strain to the same mispair in a *pol2–4* strain. For example, the *trp5-G148T pol3–5DV* F reversion rate (presumably occurring via A-G mispairs) is 29-fold greater than the *trp5-G148T pol2–4* R reversion rate (also dominated by A-G mispairs) (Fig. 3). These comparisons show that for the same mispair, in MMR-deficient strains, the reversion rate of the *pol3–5DV* strain is greater than the reversion rate in a *pol2–4* strain, consistent with previous results showing a greater mutator effect in Pol δ proofreading-defective strains compared to Pol ε proofreading-deficient strains. We next compared the reversion rates due to one mispair compared to the other mispair in strains with the same proofreading defect. For example, the reversion rate of a *trp5-G148T* F *pol2–4* strain (with increased T-C mispairs) is 17-fold greater than the reversion rate of the *trp5-G148T* R *pol2–4* strain (with increased A-G mispairs) (Fig. 3). In every case, the reversion rate due to increased T-C or C-T mispairs was greater than that due to increased A-G or G-A mispairs. That result suggested that either T-C mispairs were more readily formed than A-G mispairs, or that they were more easily extended, and thus more susceptible to proofreading, than A-G mispairs. This analysis helps explain the difference in mutational spectra observed in strains defective in either Pol δ or Pol ε proofreading: for at least certain mismatches, the frequency of forming and extending one mismatch is much greater than forming and extending the complementary mismatch. However, these experiments cannot distinguish whether the difference is due to likelihood of formation of a given mispair or the relative efficiency of extending a given mispair.

### Strains heterozygous for proofreading defects reveal *cis*-proofreading by Pol δ

Comparing the effect of a Pol δ proofreading defect to a Pol ε proofreading defect for the same mispair revealed that the reversion rate for *pol3–5DV msh6* mutants was 19- to 29-fold higher than for *pol2–4 msh6* mutants in *trp5-G148T* strains and approximately 2- to 7-fold higher in *trp5-A149C* strains (Fig. 3), which might suggest that Pol δ was less accurate than Pol ε in the absence of proofreading. However, an analysis of *in vitro* activity suggests that is not the case and that the accuracy of the proofreading defective enzymes is similar, with that of Pol ε being slightly less in certain instances [33]. If the inherent accuracies of Pol δ and Pol ε without proofreading activity are similar, an alternative explanation for the much higher reversion rates of *pol3–5DV msh6* strains compared to *pol2–4 msh6* strains would be that Pol δ could proofread Pol ε errors, but not vice-versa. An initial step was to ask whether a wild-type polymerase molecule was able to proofread errors created by a proofreading-defective molecule of the same type. We answered this question by constructing diploid strains deficient in MMR, but only heterozygous for proofreading defects (*msh6*/*msh6 pol2–4*/*POL2* abbreviated *pol2–4±* or *msh6*/*msh6 pol3–5DV*/*POL3*, abbreviated pol3*-5DV±*). In heterozygous strains, we would expect half of the polymerase molecules to be mutant and half to be wild-type. If errors produced by the mutant polymerase were not subject to subsequent proofreading by wild-type polymerases in the heterozygous strains, we would expect the reversion rate of the heterozygous strains to be one half that of homozygous proofreading defective strains.

**Fig 4:**
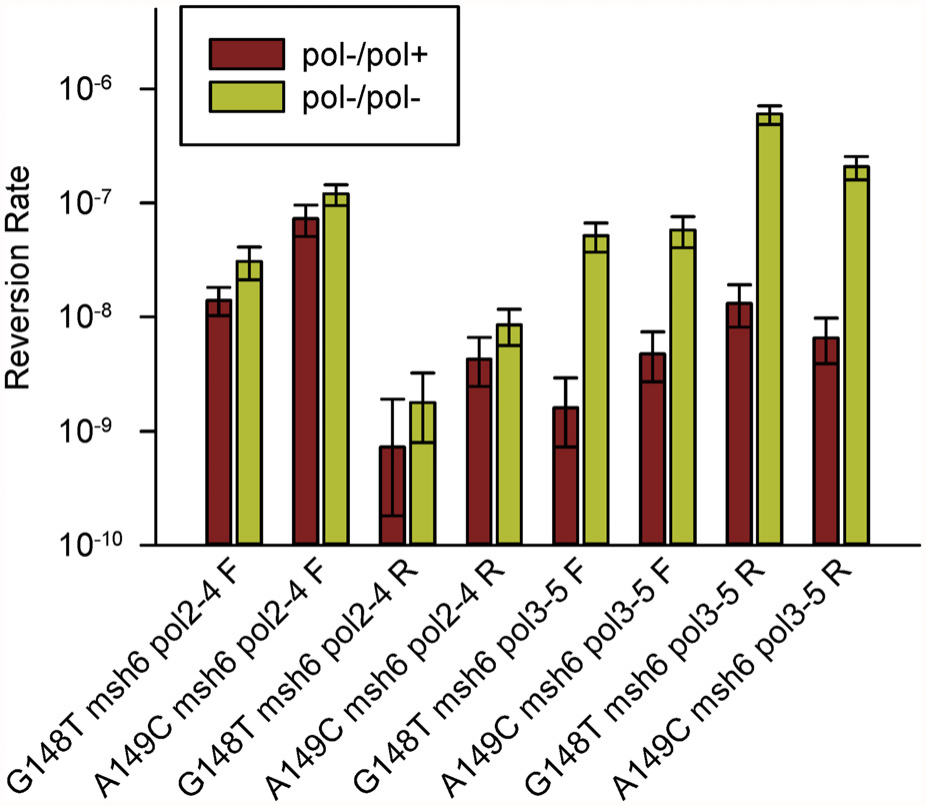
Reversion rates of heterozygous and homozygous proofreading-deficient *trp5-G148T* and *trp5-A149C* diploid strains. Shown are the reversion rates of *trp5-G148T* and *trp5-A149C* strains that are *msh6/msh6* and contain one copy of either *pol2–4* or *pol3–5DV* along with a wild-type copy of the corresponding polymerase gene (pol-/pol+). Error bars represent 95% Confidence Intervals. Also shown are the corresponding reversion rates of the homozygous proofreading-deficient strains from Fig. 2 (pol-/pol-). All data are given in S3 Table. In every case, the difference between heterozygous and homozygous is much greater in *pol3–5DV* than *pol2–4* strains.

The results are shown in Fig. 4 and S3 Table and compared in Table 1. The difference between *pol2–4±* and *pol3-5DV±* strains is striking. For Pol δ, the difference between heterozygous and homozygous strains ranged from 12- to 46-fold (Table 1). This result strongly suggests that wild-type Pol δ molecules could proofread the errors created by the proofreading-defective Pol δ molecules, as the presence of one wild-type *POL3* allele reduces reversion rates by over an order of magnitude. This conclusion is also consistent with the suggestion that Pol δ can proofread Pol α errors [10]. Proofreading of Pol α errors by Pol δ would imply that DNA strands created by Pol α, but with some error of replication, are subject to proofreading by Pol δ, as well as extension by Pol δ. Similarly, *cis*-proofreading of Pol δ errors by Pol δ would imply that a DNA strand synthesized by Pol δ but containing errors in replication would be subject to proofreading by Pol δ, as well as extension by Pol δ.

**Table 1:** Comparison of reversion rates of heterozygous and homozygous proofreading-deficient strains/

	Ratio of Reversion Rates	
	*pol2–4/pol2–4±*	*pol3–5DV/pol3–5DV±*
G148T *msh6* F	2.2*	32.
G148T *msh6* R	2.4	46.
A149C *msh6* F	1.6*	12.
A149C *msh6* R	2.0*	32.

Shown are the ratios of reversion rates from Fig. 4. For the *pol2-4/pol2–4±* comparison,

* indicates significant difference at the P = 0.05 level using 83% Confidence Intervals (S3 Table); all *pol3–5DV/pol3–5DV±* comparisons are significantly different using 95% Confidence Intervals.

Strains that were heterozygous or homozygous for Pol ε proofreading (*msh6*/*msh6 pol2–4*/*POL2* or *msh6*/*msh6 pol2–4*/*pol2–4*) were very similar in reversion rates. The increase between strains that are heterozygous in Pol ε proofreading (*pol2–4±*) and those that are homozygous was approximately two-fold for each strain (Table 1). One issue is whether that difference is statistically significant. Although it is standard to assume that 95% Confidence Intervals for two measurements should not overlap in order for the difference to be significant, in fact 95% Confidence Intervals that do not overlap are significant at the P = 0.005 level and it is 83% Confidence Intervals that are significant at the P = 0.05 level if they do not overlap [53]. Consequently, 83% Confidence Intervals were calculated for the various strains defective in Pol ε proofreading. Those results are given in S3 Table and show that three of the four comparisons in Table 1 are significantly different. A recent measurement of *CAN1* mutation rates in diploids that were *pol2–4*/*pol2–4* or *pol2–4/POL2* also found a 2-fold difference [49]. A 2-fold difference is what would be expected if a given region were replicated either by a proofreading-defective or proofreading-competent molecule and there was no compensating effect of the wild-type molecules on the proofreading-defective molecules. If the reversion rates of the *pol2–4* and *pol2–4±* strains were considered to be not significantly different, the implication would be that the *pol2–4* allele was dominant over the wild-type allele. In either case, there is no evidence of any *cis*-proofreading by Pol ε.

### *trp5-G148A* and *trp5-A149G* strains also display orientation dependent reversion rates when heterozygous for proofreading defects

**Fig 5:**
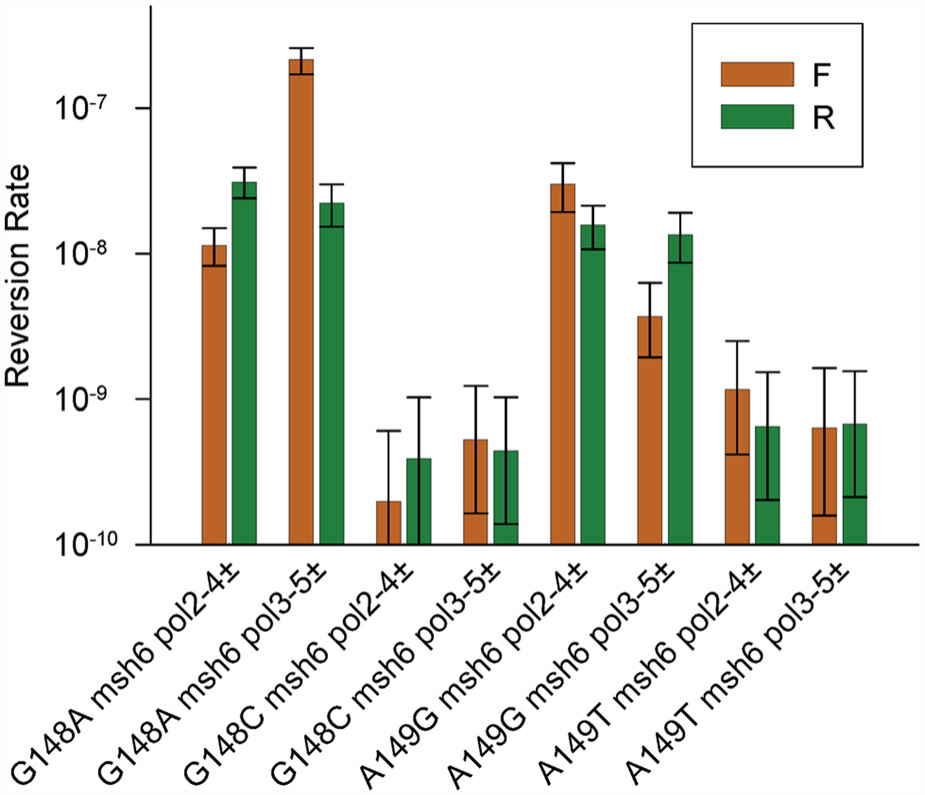
Reversion rates of additional heterozygous proofreading-deficient *trp5* strains. Shown are the reversion rates of the remaining *msh6*, heterozygous proofreading-deficient, *trp5* strains. Error bars represent 95% Confidence Intervals. Data are given in S4 Table.

**Fig 6:**
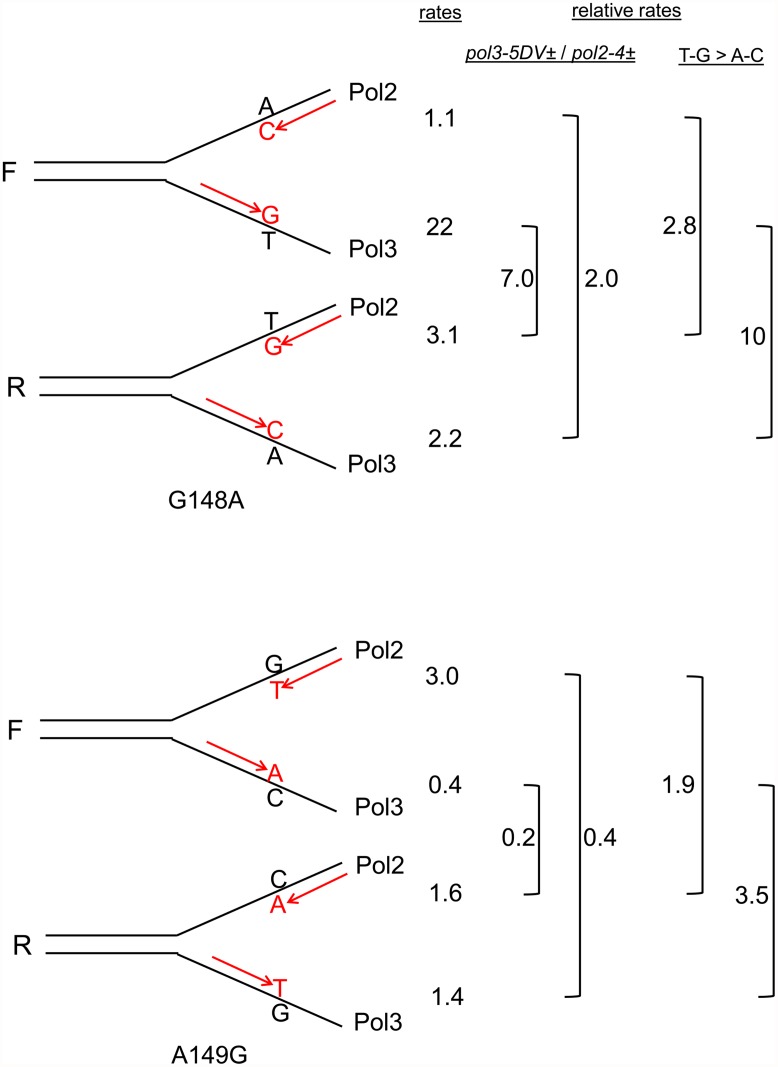
Reversion rates and expected mismatches in heterozygous proofreading-defective *trp5-G148A* and *trp5-A149G* strains. The mismatches at the replication fork necessary for reversion in the *trp5-G148A* and *trp5-A149G* strains are illustrated in the forward (F) or reverse (R) orientation relative to the *ARS306* origin of replication [37]. New strands are indicated in red, and the inserted base necessary for reversion is also indicated in red. The difference in reversion rates of the heterozygous proofreading-defective strains is due to increased mispairs on the strand replicated by the appropriate polymerase, the leading strand by Pol ε, whose catalytic subunit is Pol2, and the lagging strand by Pol δ, whose catalytic subunit is Pol3 [12]. To the right of each indicated mispair is the reversion rate of the *msh6* strain (x 10^-8^) that should have increased levels of that mispair (data from Fig. 5 and S4 Table). For example, the reversion rate of the *trp5*-*G148A* F *msh6 pol2–4±* strain is 1.1. To the right of the reversion rates are comparisons of reversion rates for the same mispair in *msh6 pol3-5DV±* versus *msh6 pol2–4±* strains and comparisons of reversion rates due to T-G compared to A-C mispairs in either *msh6 pol3–5DV±* or *msh6 pol2–4±* strains. In contrast to strains with homozygous proofreading deficiencies, the reversion rate for a given mispair in *msh6 pol3–5DV±* strains is relatively lower, or even less than, that of *msh6 pol2–4z±* strains. The reversion rate due to T-G mispairs is greater than for A-C mispairs in all cases.

We have a set of 12 *trp5* haploid strains, with 6 different alleles of *TRP5*, each in both orientations relative to the *ARS306* origin of replication [37]. An analysis of the remaining *trp5* strains defective in MMR and heterozygous in one of the proofreading mutants was performed and the results shown in Fig. 5 with the data given in S4 Table. The *trp5-G148A* and *trp5-A149G* alleles are in some ways analogous to the *trp5-G148T* and *trp5-A149C* alleles as they also revert via complementary mutations, AT→GC and GC→AT respectively (Fig. 6). However, unlike the situation with the *trp5-G148T* and *trp5-A149C* alleles, the complementary mispairs in the *trp5-A149C* strain are in the strain of opposite orientation compared to the mispairs in the *trp5-G148A* strain (compare Fig. 3 and 6). With both sets of strains, there are orientation biases in reversion rates and the biases are opposite in *msh6 pol2-4±* strains compared to *msh6 pol3-5DV±* strains (Fig. 5). The reversion rates for these strains were analyzed in Fig. 6 in a manner similar to that shown in Fig. 3 for the *trp5-G148T* and *trp5-A149C* strains. In contrast to the strains with homozygous proofreading deficiencies in Fig. 3, the relative reversion rates for a given mispair in *pol3-5DV±* compared to *pol2–4±* strains are much more similar, with the reversion rates in some *pol2–4±* strains being higher than for the equivalent mispair in *pol3-5DV±* strains (Fig. 6). In every case the loss of proofreading for a T-G mispair causes a higher reversion rate than the loss of proofreading for an A-C mispair. Thus it appears that either T-G mispairs are formed at a higher rate than A-C mispairs, or they are more easily extended.

The situation with the *trp5-G148C* and *trp5-A149T* strains is quite different. With those strains, there is a very low reversion rate even in the absence of MMR and a partial proofreading defect (Fig. 5). In all of those strains, reversion is due to the mispairing of identical bases: G-G or C-C and A-A or T-T respectively.

### Oligos creating 3′ mispairs show differential extension frequencies for different mispairs and are proofread by Pol δ but not Pol ε

Transformation of cells by single-stranded oligonucleotides (oligos) in which a permanent change is made to either chromosomal or plasmid DNA by introduction of oligos into the cell has been studied extensively in three systems: *E*. *coli*, mammalian cells, and yeast. In *E*. *coli*, numerous experiments from multiple labs support a mechanism in which oligos anneal to single-stranded DNA at the replication fork and serve as primers for continued replication, with oligos annealing to the lagging strand being considerably more efficient than when annealing to the leading strand of replication [54–62]. Mechanistic studies of oligo transformation in mammalian cells are more difficult than in *E*. *coli*. However, multiple labs have shown that oligo transformation is associated with cellular replication [63–65], that it is more efficient in S phase [66,67], that the oligo is incorporated into the genome, likely during replication [68], and that evidence suggests that the transforming oligos do so by serving as primers for replication [69–71]. Transformation in both *E*. *coli* and mammalian cells is inhibited by MMR, in agreement with the association of oligo transformation and replication [56–58,60,61,63,69,72–81]. In yeast, we have shown that oligos transform more efficiently when directed to the lagging strand [25,39,82–84], that transformation is inhibited by MMR by removing oligo sequences creating MMR-recognized mispairs [25,39,82–84], that oligos transform by incorporation [83], and that the 5’ end of transforming oligos is usually removed by a process partially dependent on Rad27, suggesting removal as part of Okazaki-like processing [84]. We also showed that in normally growing cells, MMR specifically removes oligo sequences that are part of mispairs, but that if oligo sequences escape MMR recognition and survive past S phase, MMR no longer can distinguish between the oligo and chromosomal sequences [83].

There remain two questions about oligo transformation in yeast: how oligo-directed replication could occur on the leading strand and whether transformation might generally occur in single-stranded gaps remaining in the G2 cell cycle phase. Work from the Marians lab has shown *in vitro* in *E*. *coli* that there can be “lesion skipping” on the leading strand that can result in repriming of replication [85,86]. Many years ago, it was found in UV-irradiated yeast cells that on both the leading and lagging strands short single-stranded gaps were observed that were proposed to be the result of repriming events [87]. Proposals that Pol δ could replicate the leading strand under conditions of dysfunction [19] or could be part of an alternative fork formed after stalling on the leading strand [20] would also suggest some type of repriming event on what was the leading strand. A very recent study of *in vitro* yeast replication showed that Pol ε is tightly associated with the CMG helicase during leading strand synthesis but that it can periodically cycle on and off PCNA-DNA [88]. An analysis of that work suggested that such cycling could provide access to a mismatched primer for extrinsic proofreading [89]. An oligo bound to the leading strand might appear much like a normal replicative end exposed by a cycling off of the Pol ε-CMG complex. Although we cannot rule out the possibility of transformation occurring in single-stranded gaps left in G2, we consider that possibility unlikely as a general mechanism. Our cells are undamaged and growing in rich medium before transformation. It seems unlikely that there would be sufficient single-stranded gaps in the particular region to be transformed to account for the high transformation frequencies we have observed in some cases [39]. It is also not clear why in G2 there would be five-fold or more single-stranded gaps on what used to be the lagging strand compared to what used to be the leading strand of replication. The very active involvement of MMR and of Rad27 also seem more compatible with a process occurring during replication rather than post-replicationally. Therefore we consider it likely that in yeast, as appears to be the case in *E*. *coli* and mammalian cells, oligos transform by annealing to a single-stranded region at the replication fork, with a strong preference for lagging strand, and then serve as pseudo-Okazaki primers for replication.

**Fig 7:**
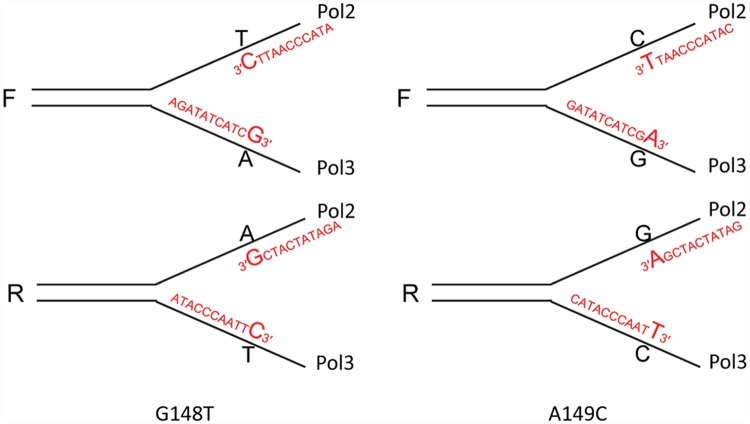
Reversion of *trp5*-G148T and *trp5*-A149C strains with oligos creating a 3′ mismatch. This diagram is similar to Fig. 3, except that the mismatch necessary for reversion of the strains is created by an oligo that forms the necessary mismatch with its 3′ terminal nucleotide. The strains will revert only if that mismatched base is extended. The oligos used were 40 nt in length; only 11 nt at the 3′ end of each oligo are shown.

If oligos can serve as primers for replication, it might be possible to transform strains with oligos that create a mispair necessary for reversion at their very 3′ end as indicated in Fig. 7 assuming the mispair was extended rather than being proofread. We tested this hypothesis by transformation using Oligo 148C with a 3′ C that would create a T-C mispair necessary for reversion of the *trp5-G148T* allele (Fig. 7). The results are given in Table 2. Because the transformation results in the table are relative to transformation with an oligo creating a mispair internal to the oligo, low transformation in these experiments indicates either the removal of the 3′ terminal mispair necessary for reversion of the strain, or failure to extend the mispair. When Oligo 148C was transformed into strains in the R orientation, which would put the oligo on the lagging strand, we obtained a relatively low number of transformants in an *msh6* strain. That number did not increase in *pol2–4* strains, but increased about 6-fold in the *msh6 pol3-5DV±* strain and about 30-fold in the *msh6 pol3–5DV* strain. In strains with the F orientation, transformation of the *msh6* strain is even lower, as the oligo would anneal to the leading strand. There is little if any increase in the *msh6 pol2–4* strain or the *msh6 pol3-5DV±* strain but a large increase (~30-fold) in the *msh6 pol3–5DV* strain. When we attempted to perform the same experiment with Oligo 148G, creating a G-A mispair on the opposite strand from Oligo148C, we obtained essentially no revertants in any background.

**Table 2:** Transformation with oligos creating mismatches at 3′ termini.

	Oligo 148C, Relative Transformation			
	*msh6*	*msh6 pol2–4*	*msh6 pol3–5DV±*	*msh6 pol3–5DV*
G148T F (Leading)	0.01±0.00	0.04±0.00	0.08±0.07	2.3±1.5**
G148T R (Lagging)	0.25±0.23	0.20±0.01	1.3±0.7	7.0±4.1*

	Oligo 148oxoG, Relative Transformation			
	*msh6*	*msh6 pol2–4*	*msh6 pol3–5DV±*	*msh6 pol3–5DV*
G148T F (Lagging)	1.1±0.2	0.45±0.2	1.4±0.7	0.89±0.34
G148T R (Leading)	2.9±1.6	3.4±2.3	2.1±1.2	1.7±0.6

	Oligo 149A, Relative Transformation			
	*msh6*	*msh6 pol2–4*	*msh6 pol3–5DV±*	*msh6 pol3–5DV*
A149C F (Lagging)	0.00	0.00±0.00	0.05±0.02	0.35±0.09
A149C R (Leading)	0	0.01±0.01	0.01±0.00	0.54±0.26**

	Oligo 149T, Relative Transformation			
	*msh6*	*msh6 pol2–4*	*msh6 pol3–5DV±*	*msh6 pol3–5DV*
A149C F (Leading)	0	0.05±0.04	0.02±0.01	0.92±0.41**
A149C R (Lagging)	0.03	0.02±0.01	0.41±0.11	2.70±1.70**

For each genotype, the average of the ratio of the number of colonies obtained with a primer creating a 3′ mismatch to a primer with an internal mismatch (Trpwt40) is shown. Note that because the efficiency of transformation with Trpwt40 varies with orientation (it anneals to the lagging strand in strains of the F orientation), one cannot compare oligo transformation of F to R strains in the above table. Note that all strains are diploid; *msh6*/*msh6 pol3–5DV/POL3* is represented as *msh6 pol3-5DV±*. There was not a significant number of transformants obtained with Oligo 148G in the *trp5-G148T* strains.

**Indicates a significant difference in transformation between *msh6 pol3–5DV* and *msh6 pol3–5DV±* strains as determined by a Mann-Whitney rank sum test.

* Indicates a significant difference in transformation between *msh6 pol3–5DV* and *msh6* strains.

A 0 in the table indicates that no revertants were observed;

0.00 indicates that revertants were observed, but fewer than 1/100 of the number seen in the internal control transformation.

We performed the same type of oligo transformation experiment in *trp5-A149C* strains, using Oligos 149A and 149T. As illustrated in Fig. 7, these oligos produce the same mismatches for extension as in the *trp5-G148T* strains, but with the opposite base as primer in the mispair. The results of these experiments are given in Table 2. As in the *trp5-G148T* strains, there is little transformation of *msh6* or *msh6 pol2–4* strains. In contrast to the results with Oligo 148G, there is measurable transformation of Oligo 149A in *msh6 pol3–5DV±* strains and substantial transformation in *msh6 pol3–5DV* strains, even when directed to the leading strand. It thus appears, at least in this sequence context, that extension of an A in a G-A mispair is much more likely than the G in an adjacent G-A mispair. Oligo 149T gives robust transformation in *msh6 pol3–5DV* strains, similar to transformation with Oligo 148C.

These experiments help make several important points. None of the oligos (Oligo 148C, Oligo 148G, Oligo 149A, or Oligo 149T) showed much transformation in *msh6* cells, but 3 of the oligos (all except Oligo 148G) showed significant transformation in *msh6 pol3–5DV* cells, when targeted to either the lagging or leading strand. Those results demonstrate that the lack of transformation in *msh6* cells is due to proofreading of the 3′ terminal mismatch by Pol δ, as elimination of Pol δ proofreading is sufficient to enable robust transformation by the oligos. In addition, the effect of Pol δ proofreading is observed, whether the oligos are targeted to the lagging or leading strand. Because incorporation of the mismatch created by the 3′ terminal base of the oligos is necessary for transformation, these results also strongly suggest that the oligos must be serving as primers for continued DNA synthesis for it is difficult to propose another mechanism that could explain oligo transformation with a 3′-terminal mismatch.

There is marked variability in the efficiency by which the oligos are able to transform. Oligo 148G gave essentially no transformants in any strain (although the same oligo with a modified G at the end gave robust transformation, see below). Oligos 148C and 149T gave the highest levels of transformation, while Oligo 149A gave markedly lower levels of transformation. These relative transformation efficiencies are similar to the differences in reversion rates seen in the *trp5-G148T* and *trp5-A149C* strains (Figs. 1 and 2; S1 and S2 Tables). In those double mutant strains, the lowest reversion rates were due to G-A mispairs in which G was on the primer strand; reversion rates due to A-G mispairs with the A on the primer strand were also very low. In all cases, reversion rates due to T-C or C-T mismatches were much higher. In our analysis of the orientation effects on reversion rates, we were not able to discriminate between reversion rate effects due to different frequencies of formation of certain mispairs or differences in elongation frequencies (see above). However, these results with oligo transformation suggest that the biased reversion rates are due at least in part to differential frequencies in elongation of various mispairs. Our oligo results show that at least in this sequence context a G paired opposite an A is very rarely elongated so that no matter how frequently such a mispair might be formed, it would rarely be extended. These results also are in line with *in vitro* mismatch extension experiments (see Discussion).

In contrast to the effects of Pol δ proofreading on oligo transformation, we observed no effect of elimination of Pol ε proofreading, whether oligos were targeted to either the leading or lagging strand. These results indicate that, no matter what mechanism is responsible for oligo transformation, it is Pol δ alone that interacts with, and elongates, the oligo. If one accepts a model in which oligos transform by priming at the replication fork, these results would suggest that any replication restart due to oligo priming on the leading strand would be extended by Pol δ and not Pol ε, in line with a model of replication restart on the leading strand being due to Pol δ [6].

We also examined oligo transformation in strains heterozygous for Pol δ proofreading (*msh6*/*msh6 pol3–5DV/POL3* or *msh6 pol3-5DV±*). As can be seen in Table 2, in most cases the difference between transformation in *msh6 pol3–5DV* strains was significantly greater than in *msh6 pol3-5DV±* strains, and in 3 cases was 20–50 fold greater. Those differences between *pol3-5DV±* and *pol3–5DV* strains are similar to the differences in reversion rates shown in Table 1 and are consistent with *cis*-proofreading by wild-type Pol δ.

These oligo transformation experiments can also help explain how one could understand proofreading by wild-type Pol δ of errors made by proofreading-defective Pol δ molecules. When a proofreading-defective Pol δ molecule inserts a mispaired base, presumably there is some frequency at which the polymerase will extend the mispair; when that happens, the mispaired base is no longer susceptible to proofreading. Frequently, one would assume that the mispaired base would stall the polymerase synthesis and in the absence of the ability to proofread, might cause a release of the polymerase, exposing the mispaired base to other exonucleases in the cell. A wild-type Pol δ molecule could bind to the primer-DNA substrate and either extend, or more likely proofread, the mispair. A proofreading-defective Pol δ molecule could interact with the substrate, either extending the mispair, or disassociating. The oligos with 3′ mispairs mimic a dissociated primer-DNA complex. In *pol3-5DV±* strains, if a proofreading-defective Pol δ molecule would usually extend the mispair when it interacted with the 3′ mispair, one would expect that the difference in extension frequencies between *pol3-5DV±* and *pol3–5DV* strains would be 2-fold. The fact that it is much greater suggests that many of the proofreading-defective polymerase interactions are not productive, allowing more chances for the mispair to be proofread by the wild-type Pol δ. This same scenario *in vivo* could explain how mispairs could be *cis*-proofread and also why there is such a large difference in reversion rates in *pol3-5DV±* and *pol3–5DV* strains.

### An oxoG-A mispair is not subject to proofreading

It is known that incorporation of oxidatively damaged nucleotides can lead to mutations [90] and that MMR can recognize 8-oxoG-A mispairs [83,91–93]. It is not known to what extent an 8-oxoG-A mispair due to incorporated 8-oxoGTP might be subject to proofreading. We therefore used Oligo 148oxoG that creates an oxoG-A mispair at the 3′ end of the oligo in the *trp5-G148T* strains. The results of those transformations are given in Table 2. The results with Oligo 148oxoG are very different from those observed with any of the other oligos. There are a substantial number of transformants in *msh6* strains of both orientations. However, there is not a significantly greater number of revertants in any proofreading-defective strain, suggesting that the oxoG-A mispair is not subject to proofreading. Because the Oligo 148oxoG has exactly the same sequence as the Oligo 148G except for the modified 3′ terminal base, these results support the conclusion that the extremely low numbers of Oligo 148G transformants are due to failure to extend the G-A mispair and not due to low formation of the G-A mispair. There is also no difference in Oligo 148oxoG transformants in *msh6 pol3-5DV±* compared to *msh6 pol3–5DV* strains in contrast to the differences in those strains observed with the other oligos. Those results suggest that there is no inherent defect in elongation ability of the proofreading defective Pol δ enzyme.

## Discussion

Our results presented above support a model in which 1) proofreading errors are usually corrected by MMR 2) in the absence of proofreading the incorporation of a mispaired base strongly depends on the efficiency of its extension by DNA polymerase; 3) upon insertion of a mispaired base, proofreading-defective DNA polymerase molecules will either extend the mispair, or failing extension will dissociate from the primer end allowing proofreading of the mispaired base by other DNA polymerase molecules; 4) DNA Pol ε is not able to proofread 3′ mispairs created by other DNA polymerase molecules including other Pol ε molecules; and 5) DNA Pol δ can proofread 3′ mispairs on both the lagging or leading strand. Not only does this model explain our results in yeast, but it can also help explain many features of tumor formation in mammals due to DNA polymerases defective in proofreading function.

### Proofreading errors are usually corrected by MMR

Some of the earliest work on proofreading and MMR in yeast found a multiplicative relationship between mutants defective in proofreading and MMR and those results were interpreted as demonstrating serial action of proofreading and MMR [3,7,94]. Our single-mutant rates, given in Fig. 2 and S1 and S2 Tables, are so low and have such large Confidence Intervals that they cannot be used in such calculations, but the double mutant rates are sufficiently high that they suggest synergism and not multiplicativity and thus seem at odds with the previous results. The two assays used by Morrison and Sugino [3,7,94] were forward mutation rate measurements in *URA3* and reversion of the *his7–2* frameshift allele. In both of those assays, the wild-type mutation rate was much higher than in our assay, and the mutation rate in the absence of MMR was increased by, in one of their haploid analyses, 41-fold in the *URA3* assay and 150-fold in the *his7–2* assay (Table 1 in [7]). It is thus very likely that the underlying mutation rates observed in those assays reflected errors not due to DNA polymerase proofreading defects. In contrast with the previous assays, we know in our *trp5* reversion assay not only what base pair mutation is made, but in the case of proofreading mutants what particular base-base mispair led to the reversion event. In the case of the single proofreading mutants, we know that a proofreading error leading to a reversion event and thus creating a base-base mispair should be corrected by MMR, and the slight increase in reversion rate is almost certainly due to random escape from MMR, as no repair system will be 100% efficient. The amount of escape would presumably depend in part on the particular mispair and sequence context, as MMR repair of base-base mispairs is sequence dependent [95]. The small increase in reversion rates observed in the *msh6* mutants is somewhat more complex. It is likely that some of the reversion events are due not to a failure in proofreading but rather to mispairs that results from damaged DNA, as we have previously demonstrated [96,97]. What appears to be a higher mutation rate in the *msh6* A149C strain compared to the *msh6* G148T strain, for example, could be due to mispairs involving an 8-oxoG and not due to proofreading errors. Such errors should not be increased in strains that would be defective in proofreading. Therefore in any proofreading-defective MMR-defective double mutant, we would expect to see a large increase in reversion rate due to the failure of MMR to repair proofreading errors, and that is what is observed.

### Mispairs are extended with variable efficiency

In the absence of MMR, the probability of a base pair mutation is a function of the probabilities of misinsertion of a base, its removal by proofreading, and elongation from the mismatched base pair. As noted above, we found a very strong orientation dependence in MMR-deficient, proofreading-defective, strains with four of the six different *trp5* mutations (*trp5-G148T*, *trp5-A149C*, *trp5-G148A*, and *trp5-A149G*; Figs. 2 and 5). However, our reversion data did not allow us to discriminate between mispairs that are formed at a high rate and mispairs that are easily extended. For example, a mispair that was easily formed, but very poorly extended, would likely contribute little to the overall reversion rate.

Our oligo transformation experiments, however, allow us to analyze efficiencies of mispair elongation. Using oligos that formed terminal A-G or C-T mispairs, we found that transformation efficiencies in *msh6 pol3–5DV* strains were quite variable depending on the particular mispair and that the variability correlated with the variability in reversion rates in *msh6 pol3–5DV* strains. Thus our results with oligos containing 3′ mismatches suggest that at least part of the reason for orientation bias in reversion rates was due to differential extension rates from various mispairs. Although as discussed above our oligo results are likely to reflect extension only by Pol δ, the fact that we see similar orientation bias with Pol ε proofreading mutants (Figs. 2 and 5), strongly suggests that Pol ε has similar elongation biases. Our oligo experiments studied only a subset of possible base mispairs—those for which we had reversion data in homozygously-deficient proofreading strains. As shown in Fig. 5, we found evidence using heterozygously-deficient proofreading strains that other mispairs also showed biased orientation effects. We think it likely that those biases could also be explained by differential mispair elongation efficiencies.

It has been difficult to devise experiments that would measure mispair extension within the context of a chromosome, as both proofreading and MMR are very effective at eliminating extensions of mispaired bases. However, there have been some *in vitro* measurements of mispair extension. Even those measurements are complicated by the demonstrated sequence effects on mispair extension [35] and the necessity to use DNA polymerases devoid of proofreading activity. For the *E*. *coli* exonuclease-deficient Klenow fragment of Polymerase I, it was found with two exceptions that in each sequence context, extension of mispairs with identical base pairs was the least favored of all combinations [35], in line with the low reversion rates of the *trp5*-G148C and *trp5*-A149T strains observed in Fig. 5. The two mispairs that were least favored of all were extension of G opposite template A and extension of A opposite template G [35], again agreeing with the failure of Oligo 148G to transform *trp5*-G148T strains and the relative low transformation of *trp5*-A149C strains with Oligo 149A (Table 2). A similar pattern of mispair extension was observed with *Taq* DNA polymerase [34] and AMV reverse transcriptase and *Drosophila* Pol α [98]. In these publications, the mispair most efficiently extended was primer G against template T [35,98], and of all of our heterozygous reversion rates, two of the three highest were mispairs in which primer G against template T would have been on the strand with a heterozygous proofreading deficiency (*trp5-G148A* F *msh6 pol3-5DV±* and *trp5-G148A* R *msh6 pol2–4±*, Fig. 5). Thus the existing *in vitro* data show clear differences for DNA polymerase extension of different 3′ terminal mispairs. Our *in vivo* results, including both reversion rates of different *trp5* mutants and our oligo transformation experiments, show biases that are consistent with the *in vitro* data demonstrating differential extension efficiencies of various mispairs. In strains proficient in proofreading, these differential extension frequencies are unlikely to be evident; however, in proofreading-defective strains, mispair extension bias is likely to be much more important, and underappreciated. Several studies have shown that the mutation spectra of Pol ε and Pol δ proofreading deficient strains differ [7,8,17]; one would expect that mispairs less likely to be extended would be most susceptible to *trans*-proofreading. Therefore we propose that differential mispair extension frequencies can explain not only the biased reversion rates we have found, but, more generally, the differences in mutation spectra observed in strains deficient in Pol δ compared to Pol ε proofreading.

### An 8-oxoG-A mispair is not subject to proofreading

Many base pair mutations are likely due to mispairings involving a damaged base. Indeed, we speculate that the higher spontaneous mutation rates in the *trp5-A149C* wild type and *msh6* strains compared to the equivalent *trp5-G149T* strains (Fig. 1) is due to oxidative damage of the template G in the *trp5*-A149C strains; we have shown that increased endogenous oxidative damage leads to greatly increased reversion rates of this mutation [96]. It is also known that incorporation of oxidized nucleotides represents a mutagenic threat to organisms [90,99] and we have previously shown that yeast can use exogenously added 8-oxoGTP and mutagenically insert it into the genome [83]. We also showed that MMR greatly inhibits the incorporation of 8-oxoGTP into the chromosome [83]. However, it has not been clear how well an 8-oxoG-A mispair could be proofread. Our interest in using Oligo 148oxoG for transformation is that it mimics the incorporation of 8-oxoGTP into the DNA and thus allows analysis of processes acting on the 8-oxoG-A mispair. In contrast to the lack of transformants with Oligo 148G, we obtained large numbers of transformants using Oligo 148oxoG, containing an 8-oxoG at the 3′ end rather than a G. As seen in Table 2, we find that the 8-oxoG-A mispair is essentially not recognized by proofreading, as there is a large number of transformants in *msh6* strains, and the number is not increased in proofreading-defective strains. Thus not only is the 8-oxoG-A mispair extended well, in stark contrast to the lack of extension of the G-A mispair, but it is not recognized by proofreading.

### *Trans* proofreading by Pol δ but not by Pol ε

It now appears well established that in yeast, and likely most eukaryotes, the leading and lagging strands of replication are usually replicated by different DNA polymerases as suggested nearly two decades ago [7] and more recently demonstrated in detail [11–13]. With that understanding, it has been difficult to explain why there is a much greater increase in the mutation rates of strains with a proofreading deficiency in Pol δ compared to Pol ε [6–8,16–18]. That result is even more surprising given evidence that MutSα function is more efficient on the lagging strand which would be replicated by Pol δ [38] and that MMR in general appears to balance the fidelity of replication of leading and lagging strands [100]. Our analysis of reversion rates in homozygously versus heterozygously proofreading-deficient strains indicated that wild-type Pol δ polymerases could *cis*-proofread, whereas wild-type Pol ε polymerases could not proofread errors created by proofreading-deficient Pol ε molecules.

Our oligo transformation experiments were consistent with the reversion analysis: wild-type Pol δ molecules prevented transformation by oligos, even in the presence of proofreading-defective Pol δ molecules. Elimination of all Pol ε proofreading activity made no difference in oligo transformation, even if the transforming oligos were targeted to the leading strand of replication. Although as stated above we cannot conclusively rule out that oligo transformation events might take place post-replicationally, the oligo transformation experiments are consistent with trans-proofreading by Pol δ, as there was insignificant transformation by oligos directed to the leading strand unless Pol δ proofreading was inactivated. It should be noted that an earlier analysis of Pol δ and Pol ε replication proposed that stalled leading strand replication would be continued by Pol δ [6].

How does a polymerase error become susceptible to proofreading by a different polymerase molecule? Because of the large size of the DNA polymerase molecules, it is likely that an error would have to cause a replication stall followed by at least partial release of the polymerase before a 3′ mispair could be exposed to a different polymerase molecule. The lagging strand is discontinuously replicated so it is not surprising that Pol δ molecules could proofread errors created by proofreading-defective Pol δ molecules, particularly as there is already evidence that Pol δ can proofread Pol α errors [10]. The leading strand is normally synthesized continuously; as noted above there is recent evidence suggesting that a Pol ε-CMG helicase complex can periodically cycle on and off PCNA-DNA and thus expose a mispair to extrinsic proofreading [88]. However, there is no known mechanism in which a different Pol ε molecule could be brought in to proofread, which is consistent with our reversion analysis demonstrating lack of Pol ε cis-proofreading. Our oligo transformation experiments suggest an additional possibility: that Pol δ molecules could proofread errors on the leading strand.

Given the possibility of Pol δ proofreading of Pol ε errors, we can examine our reversion data for evidence of such *trans*-proofreading and how extensive it might be. The increase in reversion rate in *msh6 pol2–4* strains by orders of magnitude over either single mutant indicates that a large number of Pol ε errors are not subject to Pol δ proofreading. Those reversion events must be due to errors by the proofreading-defective Pol ε polymerase that did not cause polymerase dissociation but were then extended by the polymerase. For Pol δ, there is a large increase in reversion rates, not only of *msh6 pol3–5DV* strains compared to either single mutant, but of *msh6 pol3–5DV* strains compared to *msh6 pol3-5DV±* strains (Table 1). The reversion rate in the *msh6 pol3–5DV* strains is due to the error rate of the *pol3–5DV* enzyme. Assuming that half of the replication in the *msh6 pol3-5DV±* strains is done by the wild-type Pol δ and half by the proofreading defective Pol δ, we would expect the reversion rate in the *msh6 pol3-5DV±* strains to be one half that of the *msh6 pol3–5DV* strains. The fact that the difference is 12- to 46-fold indicates that more than 90% of the time, a polymerase error results in a polymerase dissociation event that allows proofreading by a wild-type Pol δ enzyme. We can then assume that the reversion rate in a *pol3-5DV±* strain is indicative of the error rate due to molecules that do not dissociate but continue replication from the mispair. The reversion rate in *msh6 pol2–4* or *pol2–4±* strains would be a combination of errors created by Pol ε proofreading-defective molecules that did not dissociate minus errors that were created and then proofread by the wild-type Pol δ molecules.

Table 3 shows a comparison of the *msh6 pol2–4±* reversion rates compared to those of the *msh6 pol3-5DV±* strains for orientations that would have the same mispaired bases for reversion. (For example, the *trp5-G148T msh6 pol2–4± F* strain would be expected to revert via extension of a mismatched C opposite T on the leading strand, whereas the *trp5-G148T msh6 pol2–4± R* strain would be expected to revert via extension of a mismatched C opposite T on the lagging strand as illustrated in Fig. 3.) In these comparisons, the *pol2–4±* reversion rates are generally similar to the *pol3-5DV±* reversion rates. Given our assumption that each of these rates is due mainly to mispairs that are extended by the initiating polymerase, these results indicate that the underlying inaccuracy and tendency to extend mispairs of each polymerase is roughly similar. However, if one does the same comparison with the completely homozygous *msh6 pol2–4* and *msh6 pol3–5DV* strains for which we have data, the reversion rate for each mispaired base configuration is much higher with the *pol3–5DV* mutant than the *pol2–4* mutant (Table 3). As noted above, there is no reason to think that Pol ε is inherently more accurate, especially since *in vitro* results suggest that if anything Pol ε is slightly less accurate [33]; therefore the lower reversion rate for *msh6 pol2–4* strains on identical mismatches compared to *msh6 pol3–5DV* suggests that there is quite substantial Pol δ proofreading of Pol ε errors. These results also indicate that when a DNA polymerase incorporates a mispair that it is unable to proofread, there is a high probability of polymerase dissociation from the template.

**Table 3:** Comparison of reversion rates due to equivalent mismatches in *pol2–4* and *pol3–5DV* strains.

			Ratio of reversion rate	
	Template base	Primer base	*pol3-5DV±* /*pol2–4±*	*pol3–5DV*/*pol2–4*
G148T *msh6 pol2–4* F	T	C	0.9§	19
G148T *msh6 pol3–5* R	T	C		
G148T *msh6 pol3–5* F	A	G	2.2§	29
G148T *msh6 pol2–4* R	A	G		
A149C *msh6 pol2–4* F	C	T	0.1	1.8
A149C *msh6 pol3–5* R	C	T		
A149C *msh6 pol3–5* F	G	A	1.1§	6.8
A149C *msh6 pol2–4* R	G	A		
G148A *msh6 pol2–4* F	A	C	2.0	
G148A *msh6 pol3–5* R	A	C		
G148A *msh6 pol3–5* F	T	G	7.0	
G148A *msh6 pol2–4* R	T	G		
A149G msh6 pol2–4 F	G	T	0.4	
A149G msh6 pol3–5 R	G	T		
A149G msh6 pol3–5 F	C	A	0.2	
A149G msh6 pol2–4 R	C	A		

Reversion rate data are from Figs. 2, 4 and 5. Reversion is assumed to be due to the mismatch created on the strand replicated by the mutant polymerase.

§ Indicates that ratio is not significantly different from 1.

Some results in the literature have been used to suggest that there could be functional redundancy of Pol δ and Pol ε proofreading activities so that Pol ε proofreading might, for example, be able to correct Pol δ errors. For example, a study of proofreading and MMR using a frameshift reversion assay found reversion rates of triple mutants (*pol2–4 pol3–01 msh2*) were not higher than that of double mutants and found two hotspots in mutation spectra of *pol2–4 pol3–01* double mutants that were not present in either single mutant [17]. Based on those results, the authors suggested “The presence of these hotspots only in the double mutant is consistent with the functional redundancy between the Pol δ and Pol ε exonuclease activities as deduced from mutation rate measurements.” [17]. Comparison of mutation rates in very sick strains is quite problematic. Our double mutant strains that were only partially deficient in MMR (*msh6*) were quite sick; the strains used in the cited experiments were completely deficient in MMR (*msh2*). The phenomenon of “error catastrophe” and saturation of MMR was shown in *E*. *coli* in 1996, for example [101] and is likely observed in those results, as the measured mutation rate of a *pol2–4 pol3–01 msh2* strain is actually lower than that of a *pol2–4 pol3–01* strain [17]. Because of those high mutation rates one would assume that MMR would be reduced due to saturation in the *pol2–4 pol3–01* strain. Therefore hotspots that would appear in the double mutant strain could be due to mispairs that were less well recognized by MMR and would be more likely to escape in the partially MMR-defective environment of the double mutant strain. In addition, it is now known that there are suppressors that can arise in mutants that are defective in MMR and proofreading [40,48], which is one reason we have been careful to use multiple isolates in our experiments. A proposed functional redundancy of the proofreading exonucleases would be counter to the finding that Pol ε cannot even *cis*-proofread its own errors, and more importantly could not explain the much higher mutation rates that have been consistently observed in Pol δ proofreading-defective strains compared to Pol ε proofreading-defective strains.

Our results demonstrating *trans*-proofreading by Pol δ are also consistent with experiments that show that MMR appears to use Pol δ for resynthesis of DNA on either replication strand after mismatch excision [18,22,23,41,102–105]. In MMR, extension of the newly excised primer strand would be analogous to extension of one of our oligos. The disassociation of the polymerase from certain mispairs could also explain the replication checkpoint activation seen in Pol δ proofreading defective strains [46]; once a poorly-extended mispair is incorporated in a Pol δ proofreading defective strain, replication would be inhibited. Because of the low mutation rate of a *pol2–4* mutant in their assay [46], it was not possible to determine if there were a replication checkpoint activation due to Pol ε proofreading defective mutations, but our results would suggest that checkpoint activation would not be observed, due to proofreading of the Pol ε errors by wild-type Pol δ molecules.

### Replication by Pol δ and Pol ε

In order to explain experimental results that were not consistent with a model of replication in which Pol δ was responsible for all lagging strand synthesis and Pol ε was responsible for all leading strand synthesis, Pavlov and Shcherbakova proposed that lagging strand synthesis was performed by Pol δ, but that leading strand synthesis, although begun by Pol ε, was completed after any interruption by Pol δ [6]. Their model proposes that the lower mutation rate of strains lacking Pol ε proofreading relative to those lacking Pol δ proofreading can be explained by the fact that switching of leading strand synthesis to Pol δ is the rule, and “the majority of the genome replication involves copying of both DNA strands by Pol δ” [6]. That model, however, is inconsistent with recent whole genome sequence analysis of replication in yeast by fidelity mutants of Pol α, Pol δ, and Pol ε indicating that most leading strand synthesis must be done by Pol ε [106]. Our model differs from that of Pavlov and Shcherbakova in that Pol δ synthesis on the leading strand could be accompanied by proofreading of Pol ε errors, thus substantially reducing the amount of leading strand synthesis by Pol δ necessary to explain the differential mutation rates observed in proofreading-defective strains. Thus our model can explain the considerably higher mutation rate of strains deficient in Pol δ proofreading compared to Pol ε proofreading, the lower viability of such Pol δ strains, but also the observation that each polymerase is responsible for most replication of only one strand of DNA.

### Proofreading mutations and cancer

We chose to analyze proofreading mutations in the absence of MMR due to the known synergism of proofreading mutations and MMR and also because of the very low reversion rates in our strains, even when defective in proofreading. In principle, one would expect that any errors normally corrected by proofreading would be repaired by MMR. However, the fact that defects in proofreading alone do show increased mutation rates [7] is an indication that some of the excess replication errors manage to escape MMR. Recently, mutations in Pol ε and Pol δ in human endometrial and colorectal cancers have been found that appear to be pathogenic [49,107–110]. Although many of the mutations appear to be in domains that would affect proofreading, one of the more common mutations appears to affect fidelity as well as proofreading [49]. In general, these mutations appear to be heterozygous, inherited dominantly although somatic mutations are also seen, present in MMR proficient tumors, and the cancer spectrum of Pol δ and Pol ε mutations appears to be different [107–109]. Proofreading mutations have been made in the Pol δ and Pol ε polymerases of mice and studied *in vivo*. In contrast to the results seen in human tumors mentioned above, there is no tumor phenotype of heterozygous proofreading mutations in either Pol δ or Pol ε, but a robust tumor phenotype for homozygous mutations [111,112]. The tumor phenotype of the two proofreading mutations was perhaps even more distinct than that observed in humans [111]. The mutation rates of proofreading defects in each polymerase was measured and the mutator phenotype of a Pol ε defect was found to be greater than that of a Pol δ defect; the mutation rate of mice with homozygous defects in both Pol δ and Pol ε proofreading was not measurably greater than that of either single defect [111]. In contrast to the tumor phenotype, there was found to be an increased mutation rate in heterozygous defects in proofreading of either Pol δ or Pol ε compared to wild-type mutation rates with the heterozygous Pol ε defect giving a larger effect than that of Pol δ [111].

The above results seen in mice and humans seem puzzling in light of previous yeast work, in which the conclusion has been that defects in Pol δ proofreading are much more mutagenic than defects in Pol ε proofreading—although those conclusions were based almost entirely on homozygous proofreading defects. However, many of those results are compatible with our findings. Most mutations due to proofreading errors, particularly those that would escape MMR, would be expected to be base pair mutations, and that in fact is what is observed in mice [111]. Oncogenic and tumor suppressor mutations due to base pair mutations would be expected to be at least somewhat sequence specific and the marked orientation biases observed in our *pol2–4* and *pol3–5DV* strains indicate that the probability of a given base pair mutation could be strongly dependent on which polymerase was proofreading defective and the orientation of replication of that gene in a given tissue. Therefore the differences in tumor spectra are perhaps not so surprising. The relative prevalence of Pol ε mutations compared to Pol δ mutations in human tumors is one of the most striking differences compared to what would have been expected from previous yeast work. Most of the observed human proofreading mutations are heterozygous and as Table 3 indicates, even in the absence of MMR, the reversion rate of some of our *trp5* strains is higher or about the same level in *pol2–4±* strains compared to *pol3-5DV±* strains. It has been found that MMR due to MutSα is more efficient on the lagging strand than the leading strand, at least in yeast [38], which would tend to reduce even further the relative mutational bias in Pol δ mutants compared to Pol ε mutants. It is also possible that there could be some selective pressure for second site mutations to moderate the error rate of either proofreading polymerase as has been observed in yeast [40,48], particularly in homozygously-deficient animals. The fact that heterozygous defects in proofreading can lead to tumors in humans, but not in mice, is similar to findings with other genes and may be reflective of the much longer lifespan of humans than mice. For example, mice homozygously-deficient in *Msh6* show a strong tumor phenotype, but there is little increase in heterozygous mice [113]. In humans inheriting heterozygous *MSH6* mutations, there is a significant increase in various types of tumors [114].

## Materials and Methods

### *S. cerevisiae* strains and oligos

The genotypes of all strains used in these experiments can be found in S5 Table. All haploid strains containing a *TRP5* point mutation were derivatives of the strains previously published [37]. For creation of diploid strains that would be hemizygous for the *trp5* point mutations, we used a haploid strain of opposite mating type, BY4741 [115] that shared parentage with our strains, but contained complementary markers. We further modified BY4741 by restoring the strain to Leu+ and making an exact deletion of the *TRP5* gene by delitto perfetto [116], creating GCY2122 (S5 Table). The *MSH6* gene was deleted by transformation with a PCR fragment generated from the *MSH6* gene deletion described in [117] or from a strain containing an *MSH6* deletion created with a loxP-kanMX-loxP fragment [118]. When a second allele of *MSH6* was to be deleted in a diploid strain, a PCR fragment obtained from a strain containing an *MSH6* deletion made by insertion of the loxLE-hphNT1-loxRE fragment contained in pZC3 [119] was used. *pol2–4* haploid mutants were created by transformation with plasmid YIpJB1 as described [4]. *pol3–5DV* haploid mutants were created by transformation with *Eag*I-digested pY19 [43], selecting for Ura+ cells. Cells were subsequently selected for *URA3* loss and screening for strains containing the *pol3–5DV* mutation. In order to create *msh6 pol3–5DV* haploid strains, *pol3–5DV* cells were first transformed with pBL304, a plasmid containing *POL3* on a *URA3 CEN* plasmid, which was constructed by Peter Burgers and is described in [7]. The *MSH6* gene in such strains could subsequently be deleted with the strain maintaining viability. Diploid strains were constructed by mating of two haploid strains followed by selection on synthetic dextrose (SD) medium lacking methionine and leucine [120]. Diploid *msh6 pol3–5DV* strains were constructed by mating of the two haploids, one being *MSH6*, and the other containing the *POL3* plasmid pBL304 rescuing the *msh6 pol3–5DV* genotype, followed by deletion of the second *MSH6* allele with a *hphNT1* marker [119] followed by selection for loss of the *POL3* plasmid. Oligos used for transformation were gel purified (Eurofins MWG Operon); the sequences are listed in S6 Table.

### Reversion analysis

Reversion analysis was performed as described [96]. Reversion rates and Confidence Intervals were calculated [96] using the program Salvador [121–123]. When multiple reversion experiments were done for a given genotype, the median value was used for subsequent analysis. The reversion rates of heterozygous and homozygous proofreading-deficient strains (Table 2) were considered to be different if the 83% confidence levels did not overlap [53].

### Transformation with oligos

Oligo transformation was essentially as described previously [25,82–84]. An overnight culture of a strain was diluted 1:50 in YPAD [120], incubated with shaking at 30° to an OD_600_ of 1.3–1.5, washed twice with cold H_2_O, and once with cold 1 M sorbitol. After the final centrifugation, all solution was removed from the cells and a volume of cold 1 M sorbitol equal to that of the cell pellet added to resuspend the cells. For a typical transformation, 200 pmol of a Trp oligo was added to 200 μl of this cell suspension in a 2-mm gap electroporation cuvette, and the mixture electroporated at 1.55 kV, 200 Ω, and 25 μF (BTX Harvard Apparatus ECM 630). Immediately after electroporation, the cell suspension was added to 5 ml of YPAD, and the cells incubated at 30° with shaking for 2 h. Cells were then centrifuged, washed with H_2_O, and plated on SD medium lacking tryptophan [120] to select transformants. In order to control for some of the variability we observed in transformation efficiencies, one portion of each cell suspension was electroporated with the Trpwt40 oligo, which reverts all of the strains via a centrally-located mismatched base and is thus not subject to any proofreading effect.

## Supporting Information

S1 Table Reversion rates of G148T diploid strains in Fig. 2A. (DOCX)

S2 Table Reversion rates of A149C diploid strains in Fig. 2B. (DOCX)

S3 Table Reversion rates of heterozygous and homozygous proofreading-deficient G148T and A149C diploid strains. (DOCX)

S4 Table Reversion rates of additional heterozygous proofreading-deficient *trp5* strains. (DOCX)

S5 Table Genotypes of strains. (DOCX)

S6 Table Oligo sequences. (DOCX)
