# Supplementary material for: Replicative DNA Polymerase δ but Not ε Proofreads Errors in Cis and in Trans
Source: PLoS Genet. 2015 Mar 5;11(3):e1005049. doi: 10.1371/journal.pgen.1005049 (PMC4351087; doi:10.1371/journal.pgen.1005049)
Supplement: S1 Table — (DOCX) [file pgen.1005049.s001.docx]

| Table S1. Reversion rates of G148T Diploid Strains in Figure 2A. | | | |
| --- | --- | --- | --- |
| Genotype | Reversion Rate and 95% Confidence Intervals (x10^-10^) | |  |
| wt F (4) | 2 | (1, 5) |  |
| wt R (2) | 1 | (1, 3) |  |
| msh6 F (2) | 5 | (2, 12) |  |
| msh6 R (2) | 4 | (1, 9) |  |
| pol2-4 F (2) | 12 | (5, 24) |  |
| pol2-4 R (3) | 3 | (1, 9) |  |
| pol3-5 F (2) | 6 | (1, 19) |  |
| pol3-5 R (2) | 21 | (10, 38) |  |
| msh6 pol2-4 F (3) | 310 | (210, 410) |  |
| msh6 pol2-4 R (6) | 18 | (8, 32) |  |
| msh6 pol3-5 F (4) | 520 | (370, 670) |  |
| msh6 pol3-5 R (4) | 6000 | (4900, 7100) |  |
| The parentheses after the genotype indicate the number of different isolates that were used to measure reversion rates. The reversion rate shown is from the experiment giving the median value. | | |  |
