# Supplementary material for: Replicative DNA Polymerase δ but Not ε Proofreads Errors in Cis and in Trans
Source: PLoS Genet. 2015 Mar 5;11(3):e1005049. doi: 10.1371/journal.pgen.1005049 (PMC4351087; doi:10.1371/journal.pgen.1005049)
Supplement: S2 Table — (DOCX) [file pgen.1005049.s002.docx]

| Table S2. Reversion rates of A149C Diploid Strains in Figure 2B. | | | |
| --- | --- | --- | --- |
| Genotype | Reversion Rate and 95% Confidence Intervals (x10^-10^) | |  |
| wt F | 8 | (4, 14) |  |
| wt R | 13 | (7, 22) |  |
| msh6 F | 51 | (36, 69) |  |
| msh6 R | 22 | (12, 34) |  |
| pol2-4 F (2) | 8 | (2, 18) |  |
| pol2-4 R (2) | 10 | (4, 22) |  |
| pol3-5 F | 3 | (1, 10) |  |
| pol3-5 R (3) | 4 | (1, 13) |  |
| msh6 pol2-4 F (2) | 1200 | (950, 1400) |  |
| msh6 pol2-4 R (4) | 85 | (56, 120) |  |
| msh6 pol3-5 F (3) | 580 | (400, 760) |  |
| msh6 pol3-5 R (2) | 2100 | (1600, 2600) |  |
| The parentheses after the genotype indicate the number of different isolates that were used to measure reversion rates when more than one isolate was used. The reversion rate shown is from the experiment giving the median value. | | |  |
