# Supplementary material for: Replicative DNA Polymerase δ but Not ε Proofreads Errors in Cis and in Trans
Source: PLoS Genet. 2015 Mar 5;11(3):e1005049. doi: 10.1371/journal.pgen.1005049 (PMC4351087; doi:10.1371/journal.pgen.1005049)
Supplement: S3 Table — (DOCX) [file pgen.1005049.s003.docx]

| Table S3. Reversion rates of heterozygous and homozygous proofreading-deficient G148T and A149C diploid strains. | | | |  |
| --- | --- | --- | --- | --- |
| Genotype | Reversion Rate and 95% Confidence Intervals (x10^-10^) | | | |
| G148T msh6 pol2-4 F ± (3) | | 140 | (100, 180) | |
| G148T msh6 pol2-4 F (3) | | 310 | (210, 410) | |
| A149C msh6 pol2-4 F ± (6) | | 730 | (510, 960) | |
| A149C msh6 pol2-4 F (2) | | 1200 | (950, 1400) | |
| G148T msh6 pol2-4 R ± (5) | | 7 | (2, 19) | |
| G148T msh6 pol2-4 R (6) | | 18 | (8, 32) | |
| A149C msh6 pol2-4 R ± (4) | | 43 | (25, 66) | |
| A149C msh6 pol2-4 R (4) | | 85 | (56, 120) | |
| G148T msh6 pol3-5 F ± (2) | | 16 | (7, 29) | |
| G148T msh6 pol3-5 F (4) | | 520 | (370, 670) | |
| A149C msh6 pol3-5 F ± (2) | | 47 | (27, 74) | |
| A149C msh6 pol3-5 F (3) | | 580 | (400, 760) | |
| G148T msh6 pol3-5 R ± | | 130 | (82, 190) | |
| G148T msh6 pol3-5 R (4) | | 6000 | (4900, 7100) | |
| A149C msh6 pol3-5 R ± (2) | | 65 | (39, 98) | |
| A149C msh6 pol3-5 R (2) | | 2100 | (1600, 2600) | |
| The parentheses after the genotype indicate the number of different isolates that were used to measure reversion rates when more than one isolate was used. The reversion rate shown is from the experiment giving the median value. | | | | |
