# Supplementary material for: Replicative DNA Polymerase δ but Not ε Proofreads Errors in Cis and in Trans
Source: PLoS Genet. 2015 Mar 5;11(3):e1005049. doi: 10.1371/journal.pgen.1005049 (PMC4351087; doi:10.1371/journal.pgen.1005049)
Supplement: S4 Table — (DOCX) [file pgen.1005049.s004.docx]

| Table S4. Reversion rates of additional heterozygous proofreading-deficient *trp5* strains | | | |  |
| --- | --- | --- | --- | --- |
| Genotype | Reversion Rate and 95% Confidence Intervals (x10^-10^) | | | |
| G148A msh6 pol2-4± F (2) | | 110 | (82, 150) | |
| G148A msh6 pol2-4± R | | 310 | (240, 390) | |
| G148A msh6 pol3-5± F (2) | | 2200 | (1700, 2600) | |
| G148A msh6 pol3-5± R | | 220 | (150, 300) | |
| G148C msh6 pol2-4± F (4) | | 2 | (0, 6) | |
| G148C msh6 pol2-4± R (6) | | 4 | (1, 10) | |
| G148C msh6 pol3-5± F | | 5 | (2, 12) | |
| G148C msh6 pol3-5± R | | 4 | (1, 10) | |
| A149G msh6 pol2-4± F | | 300 | (190, 420) [220, 380] | |
| A149G msh6 pol2-4± R | | 160 | (110, 210) [120, 200] | |
| A149G msh6 pol3-5± F (3) | | 37 | (19, 63) | |
| A149G msh6 pol3-5± R | | 140 | (86, 190) | |
| A149T msh6 pol2-4± F | | 12 | (4, 25) | |
| A149T msh6 pol2-4± R | | 7 | (2, 15) | |
| A149T msh6 pol3-5± F | | 6 | (2, 16) | |
| A149T msh6 pol3-5± R | | 7 | (2, 16) | |
| Numbers in [ ] represent 83% Confidence Intervals. The parentheses after the genotype indicate the number of different isolates that were used to measure reversion rates when more than one isolate was used. The reversion rate shown is from the experiment giving the median value. | | | | |
