# Supplementary material for: Replicative DNA Polymerase δ but Not ε Proofreads Errors in Cis and in Trans
Source: PLoS Genet. 2015 Mar 5;11(3):e1005049. doi: 10.1371/journal.pgen.1005049 (PMC4351087; doi:10.1371/journal.pgen.1005049)
Supplement: S5 Table — (DOCX) [file pgen.1005049.s005.docx]

| **Table S5.** Genotypes of strains. | | | |
| --- | --- | --- | --- |
| **Strain Number** | ***TRP5* Mutation** | **Relevant Genotype** | **Reference** |
| BY4741 |  | *MAT***a** *ura3Δ0 his3Δ1 leu2Δ0 met15Δ0* | [1] |
| GCY2122 |  | *MAT***a** *ura3Δ0 his3Δ1 met15Δ0 trp5Δ* | This study |
| GCY2145 |  | GCY2122 *msh6::kanMX* | This study |
| GCY2129, GCY2678 |  | GCY2122 *pol2-4* | This study |
| GCY2430 |  | GCY2129  *lys2::hisG pol2-4* | This study |
| GCY2146 |  | GCY2129 *msh6::kanMX pol2-4* | This study |
| GCY2348 |  | GCY2122 *pol3-5DV* | This study |
| GCY2479 |  | GCY2348 *lys2::hisG pol3-5DV* | This study |
| GCY2457 |  | GCY2348 *pol3-5DV*  *[POL3]* | This study |
| GCY2927 |  | GCY2457 *msh6::* *hphNT1 pol3-5DV [POL3]* | This study |
|  |  |  |  |
| GCY1675 | A149C-F | *MATα his3Δ200 ura3-52 leu2Δ1 trp5-A149C* | [2] |
| GCY2038 | A149C-F | GCY1675 *msh6::kanMX* | [3] |
| GCY2662 | A149C-F | GCY1675 *pol2-4* | This study |
| GCY2677 | A149C-F | GCY1675 *pol3-5DV* | This study |
| GCY2736 | A149C-F | GCY2662 *msh6::kanMX pol2-4* | This study |
| GCY2242 | A149C-F | GCY1675 X GCY2122 *trp5-A149C/trp5Δ* | This study |
| GCY2250 | A149C-F | GCY2038 X GCY2145 *trp5-A149C/trp5Δ msh6*/*msh6* | This study |
| GCY2669 | A149C-F | GCY2662 X GCY2129 *trp5-A149C/trp5Δ pol2-4/pol2-4* | This study |
| GCY2707 | A149C-F | GCY2662 X GCY2678 *trp5-A149C/trp5Δ pol2-4/pol2-4* | This study |
| GCY2709 | A149C-F | GCY2677 X GCY2348 *trp5-A149C/trp5Δ pol3-5DV/pol3-5DV* | This study |
| GCY2220 | A149C-F | GCY2038 X GCY2146 *trp5-A149C/trp5Δ msh6*/*msh6 POL2/pol2-4* | This study |
| GCY2653 | A149C-F | GCY2038 X GCY2129 *trp5-A149C/trp5Δ msh6*/*msh6 POL2/pol2-4* | This study^a^ |
| GCY2791 | A149C-F | GCY2736 X GCY2145 *trp5-A149C/trp5Δ msh6*/*msh6 POL2/pol2-4* | This study |
| GCY2741 | A149C-F | GCY2736 X GCY2146 *trp5-A149C/trp5Δ msh6*/*msh6 pol2-4/pol2-4* | This study |
| GCY2655 | A149C-F | GCY2038 X GCY2348 *trp5-A149C/trp5Δ msh6*/*msh6 POL3/pol3-5DV* | This study^a^ |
| GCY2928 | A149C-F | GCY2677 X GCY2927 *trp5-A149C/trp5Δ msh6*/*msh6 pol3-5DV/pol3-5DV* | This study^a^ |
|  |  |  |  |
| GCY1649 | A149C-R | *MATα his3Δ200 ura3-52 leu2Δ1 trp5-A149C* | [2] |
| GCY2043 | A149C-R | GCY1649 *msh6::loxP-kanMX* | [3] |
| GCY2154 | A149C-R | GCY2043 *msh6::loxP* | This study |
| GCY2663 | A149C-R | GCY1649 *pol2-4* | This study |
| GCY2917 | A149C-R | GCY1649 *pol3-5DV* | This study |
| GCY2742 | A149C-R | GCY2663 *msh6::kanMX pol2-4* | This study |
| GCY2288 | A149C-R | GCY1649 X GCY2122 *trp5-A149C/trp5Δ* | This study |
| GCY2290 | A149C-R | GCY2154 X GCY2145 *trp5-A149C/trp5Δ msh6*/*msh6* | This study |
| GCY2670 | A149C-R | GCY2663 X GCY2129 *trp5-A149C/trp5Δ pol2-4/pol2-4* | This study |
| GCY2708 | A149C-R | GCY2663 X GCY2678 *trp5-A149C/trp5Δ pol2-4/pol2-4* | This study |
| GCY2918 | A149C-R | GCY2917 X GCY2348 *trp5-A149C/trp5Δ pol3-5DV/pol3-5DV* | This study |
| GCY2221 | A149C-R | GCY2154 X GCY2146 *trp5-A149C/trp5Δ msh6*/*msh6 POL2/pol2-4* | This study |
| GCY2654 | A149C-R | GCY2043 X GCY2129 *trp5-A149C/trp5Δ msh6*/*msh6 POL2/pol2-4* | This study^a^ |
| GCY2792 | A149C-R | GCY2742 X GCY2145 *trp5-A149C/trp5Δ msh6*/*msh6 POL2/pol2-4* | This study |
| GCY2743, 2814 | A149C-R | GCY2742 X GCY2146 *trp5-A149C/trp5Δ msh6*/*msh6 pol2-4/pol2-4* | This study |
| GCY2656 | A149C-R | GCY2043 X GCY2348 *trp5-A149C/trp5Δ msh6*/*msh6 POL3/pol3-5DV* | This study^a^ |
| GCY2935 | A149C-R | GCY2917 X GCY2927 *trp5-A149C/trp5Δ msh6*/*msh6 pol3-5DV/pol3-5DV* | This study^a^ |
|  |  |  |  |
| GCY1663 | A149G-F | *MATα his3Δ200 ura3-52 leu2Δ1 trp5-A149G* | [2] |
| GCY2184 | A149G-F | GCY1663 *msh6::kanMX* | [3] |
| GCY2280 | A149G-F | GCY2184 X GCY2146 *trp5-A149G/trp5Δ msh6*/*msh6 POL2/pol2-4* | This study |
| GCY2688, 2835 | A149G-F | GCY2184 X GCY2348 *trp5-A149G/trp5Δ msh6*/*msh6 POL3/pol3-5DV* | This study^a^ |
|  |  |  |  |
| GCY1638 | A149G-R | *MATα his3Δ200 ura3-52 leu2Δ1 trp5-A149G* | [2] |
| GCY2045 | A149G-R | GCY1638 *msh6::kanMX* | This study |
| GCY2687 | A149G-R | GCY2045 X GCY2146 *trp5-A149G/trp5Δ msh6*/*msh6 POL2/pol2-4* | This study |
| GCY2689 | A149G-R | GCY2045 X GCY2348 *trp5-A149G/trp5Δ msh6*/*msh6 POL3/pol3-5DV* | This study^a^ |
|  |  |  |  |
| GCY1903 | A149T-F | *MATα his3Δ200 ura3-52 leu2Δ1 trp5-A149T* | [2] |
| GCY2192 | A149T-F | GCY1903 *msh6::kanMX* | [3] |
| GCY2281 | A149T-F | GCY2192 X GCY2146 *trp5-A149T/trp5Δ msh6*/*msh6 POL2/pol2-4* | This study |
| GCY2695 | A149T-F | GCY2192 X GCY2348 *trp5-A149T/trp5Δ msh6*/*msh6 POL3/pol3-5DV* | This study^a^ |
|  |  |  |  |
| GCY1920 | A149T-R | *MATα his3Δ200 ura3-52 leu2Δ1 trp5-A149T* | [2] |
| GCY2142 | A149T-R | GCY1920 *msh6::kanMX* | This study |
| GCY2699 | A149T-R | GCY2142 X GCY2146 *trp5-A149T/trp5Δ msh6*/*msh6 POL2/pol2-4* | This study |
| GCY2712 | A149T-R | GCY2142 X GCY2348 *trp5-A149T/trp5Δ msh6*/*msh6 POL3/pol3-5DV* | This study^a^ |
|  |  |  |  |
| GCY1868 | G148A-F | *MATα his3Δ200 ura3-52 leu2Δ1 trp5-G148A* | [2] |
| GCY2037 | G148A-F | GCY1868 *msh6::kanMX* | [3] |
| GCY2219, 2228 | G148A-F | GCY2037 X GCY2146 *trp5-G148A/trp5Δ msh6*/*msh6 POL2/pol2-4* | This study |
| GCY2729 | G148A-F | GCY2037 X GCY2348 *trp5-G148A/trp5Δ msh6*/*msh6 POL3/pol3-5DV* | This study^a^ |
|  |  |  |  |
| GCY1695 | G148A-R | *MATα his3Δ200 ura3-52 leu2Δ1 trp5-G148A* | [2] |
| GCY2044 | G148A-R | GCY1695 *msh6::loxP-kanMX* | This study |
| GCY2155 | G148A-R | GCY1695 *msh6::loxP* | This study |
| GCY2214 | G148A-R | GCY2155 X GCY2146 *trp5-G148A/trp5Δ msh6*/*msh6 POL2/pol2-4* | This study |
| GCY2712 | G148A-R | GCY2044 X GCY2348 *trp5-G148A/trp5Δ msh6*/*msh6 POL3/pol3-5DV* | This study^a^ |
|  |  |  |  |
| GCY1756 | G148C-F | *MATα his3Δ200 ura3-52 leu2Δ1 trp5-G148C* | [2] |
| GCY2141 | G148C-F | GCY1756 *msh6::loxP-kanMX* | [3] |
| GCY2245 | G148C-F | GCY1756 *msh6::loxP* | This study |
| GCY2202 | G148C-F | GCY2141 X GCY2146 *trp5-G148C/trp5Δ msh6*/*msh6 POL2/pol2-4* | This study |
| GCY2282 | G148C-F | GCY2245 X GCY2146 *trp5-G148C/trp5Δ msh6*/*msh6 POL2/pol2-4* | This study |
| GCY2727 | G148C-F | GCY2141 X GCY2348 *trp5-G148C/trp5Δ msh6*/*msh6 POL3/pol3-5DV* | This study^a^ |
|  |  |  |  |
| GCY1717 | G148C-R | *MATα his3Δ200 ura3-52 leu2Δ1 trp5-G148C* | [2] |
| GCY2139 | G148C-R | GCY1717 *msh6::kanMX* | This study |
| GCY2200, 2211 | G148C-R | GCY2139 X GCY2146 *trp5-G148C/trp5Δ msh6*/*msh6 POL2/pol2-4* | This study |
| GCY2843, 2844 | G148C-R | GCY2139 X GCY2146 *trp5-G148C/trp5Δ msh6*/*msh6 POL2/pol2-4* | This study |
| GCY2728 | G148C-R | GCY2139 X GCY2348 *trp5-G148C/trp5Δ msh6*/*msh6 POL3/pol3-5DV* | This study^a^ |
|  |  |  |  |
| GCY1862 | G148T-F | *MATα his3Δ200 ura3-52 leu2Δ1 trp5-G148T* | [2] |
| GCY2036 | G148T-F | GCY1862 *msh6::kanMX* | [3] |
| GCY2506 | G148T-F | GCY1862 *lys2CT_1265_GA* | This study |
| GCY2808 | G148T-F | GCY1862 *pol2-4* | This study |
| GCY2512 | G148T-F | GCY2506 *msh6::kanMX* | This study |
| GCY2809 | G148T-F | GCY1862 *pol3-5DV* | This study |
| GCY2929 | G148T-F | GCY2808 *msh6::kanMX pol2-4* | This study |
| GCY2307 | G148T-F | GCY1862 X GCY2122 *trp5-G148T/trp5Δ* | This study |
| GCY2225 | G148T-F | GCY2036 X GCY2145 *trp5-G148T/trp5Δ msh6*/*msh6* | This study |
| GCY2854 | G148T-F | GCY2808 X GCY2678 *trp5-G148T/trp5Δ pol2-4/pol2-4* | This study |
| GCY2855 | G148T-F | GCY2809 X GCY2348 *trp5-G148T/trp5Δ pol3-5DV/pol3-5DV* | This study |
| GCY2632 | G148T-F | GCY2512 X GCY2430 *trp5-G148T/trp5Δ msh6*/*msh6 POL2/pol2-4* | This study |
| GCY2931 | G148T-F | GCY2929 X GCY2146 *trp5-G148T/trp5Δ msh6*/*msh6 pol2-4/pol2-4* | This study |
| GCY2634 | G148T-F | GCY2512 X GCY2479 *trp5-G148T/trp5Δ msh6*/*msh6 POL3/pol3-5DV* | This study^a^ |
| GCY2933 | G148T-F | GCY2809 X GCY2927 *trp5-G148T/trp5Δ msh6*/*msh6 pol3-5DV/pol3-5DV* | This study^a^ |
|  |  |  |  |
| GCY1718 | G148T-R | *MATα his3Δ200 ura3-52 leu2Δ1 trp5-G148T* | [2] |
| GCY2140 | G148T-R | GCY1718 *msh6::kanMX* | This study |
| GCY2251 | G148T-R | GCY1718 *lys2CT_1265_GA* | This study |
| GCY2810 | G148T-R | GCY1718 *pol2-4* | This study |
| GCY2253 | G148T-R | GCY2251 *msh6::kanMX* | This study |
| GCY2811 | G148T-R | GCY1718 *pol3-5DV* | This study |
| GCY2930 | G148T-R | GCY2810 *msh6::kanMX pol2-4* | This study |
| GCY2480 | G148T-R | GCY1718 X GCY2122 *trp5-G148T/trp5Δ* | This study |
| GCY2227 | G148T-R | GCY2140 X GCY2145 *trp5-G148T/trp5Δ msh6*/*msh6* | This study |
| GCY2856 | G148T-R | GCY2810 X GCY2678 *trp5-G148T/trp5Δ pol2-4/pol2-4* | This study |
| GCY2857 | G148T-R | GCY2811 X GCY2348 *trp5-G148T/trp5Δ pol3-5DV/pol3-5DV* | This study |
| GCY2633 | G148T-R | GCY2253 X GCY2430 *trp5-G148T/trp5Δ msh6*/*msh6 POL2/pol2-4* | This study^a^ |
| GCY2932 | G148T-R | GCY2930 X GCY2146 *trp5-G148T/trp5Δ msh6*/*msh6 pol2-4/pol2-4* | This study |
| GCY2635 | G148T-R | GCY2253 X GCY2479 *trp5-G148T/trp5Δ msh6*/*msh6 POL3/pol3-5DV* | This study^a^ |
| GCY2934 | G148T-R | GCY2811 X GCY2927 *trp5-G148T/trp5Δ msh6*/*msh6 pol3-5DV/pol3-5DV* | This study^a^ |

^a^After the indicated mating, the second allele of *MSH6* was deleted in the diploid strain.

REFERENCES

1. Brachmann CB, Davies A, Cost GJ, Caputo E, Li JC, et al. (1998) Designer deletion strains derived from *Saccharomyces cerevisiae* S288C: a useful set of strains and plasmids for PCR-mediated gene disruption and other applications. Yeast 14: 115-132.

2. Williams T-M, Fabbri RM, Reeves JW, Crouse GF (2005) A new reversion assay for measuring all possible base pair substitutions in *Saccharomyces cerevisiae*. Genetics 170: 1423-1426.

3. Shockley AH, Doo DW, Rodriguez GP, Crouse GF (2013) Oxidative damage and mutagenesis in *Saccharomyces cerevisiae*: genetic studies of pathways affecting replication fidelity of 8-oxoguanine. Genetics 195: 359-367.
