# Supplementary material for: Replicative DNA Polymerase δ but Not ε Proofreads Errors in Cis and in Trans
Source: PLoS Genet. 2015 Mar 5;11(3):e1005049. doi: 10.1371/journal.pgen.1005049 (PMC4351087; doi:10.1371/journal.pgen.1005049)
Supplement: S6 Table — (DOCX) [file pgen.1005049.s006.docx]

| **Table S6**. Oligo sequences | |
| --- | --- |
| Oligo | Sequence |
| Oligo 148C | GACCATCTGCAATTGGATCAGAGAAGGGCATACCCAATTC |
| Oligo 148G | ATTCTCAAGGGTTTCCAGGATGGTGGTGTAGATATCATCG |
| Oligo 148oxoG | ATTCTCAAGGGTTTCCAGGATGGTGGTGTAGATATCATC-oxoG |
| Oligo 149A | TTCTCAAGGGTTTCCAGGATGGTGGTGTAGATATCATCGA |
| Oligo 149T | GGACCATCTGCAATTGGATCAGAGAAGGGCATACCCAATT |
| Trpwt40 | ATGGTGGTGTAGATATCATCGAATTGGGTATGCCCTTCTC |
| In the oligo sequence, the base creating the reverting mismatch is underlined; for Trpwt40 the base will be either G or A, depending on the *trp5* mutation. | |
